# Supplementary material for: Synthesis of DPIE [2-(1,2-Diphenyl-1H-indol-3-yl)ethanamine] Derivatives and Their Regulatory Effects on Pro-Inflammatory Cytokine Production in IL-1β-Stimulated Primary Human Oral Cells
Source: Molecules. 2022 Jan 28;27(3):899. doi: 10.3390/molecules27030899 (PMC8840366; doi:10.3390/molecules27030899)

Supplementary Material

Synthesis of DPIE [2-(1,2-diphenyl-1H-indol-3-yl)ethanamine] derivatives and their regulatory effects on pro-inflammatory cytokine production in IL-1β-stimulated primary human oral cells

Jeongah Lim[1†], Jihyoun Seong[2†], Seunggon Jung[3], Tae-Hoon Lee[2], Eunae Kim*[4] and Sunwoo Lee[*1]

[1]Department of Chemistry, Chonnam National University, Gwangju 61186, Korea

[2]Department of Oral Biochemistry, Dental Science Research Institute, School of Dentistry, Chonnam National University, Gwangju 61186, Korea

[3]Department of Oral & Maxillofacial Surgery, School of Dentistry, Chonnam National University, Gwangju 61186, Korea

[4] College of Pharmacy, Chosun University, Gwangju 61452, Korea

[†] These authors are equally contributed.

*Correspondence: eunaekim@chosun.ac.kr(EK), Tel: +82-62-230-6375, sunwoo@chonnam.ac.kr(SL), Tel: +82-62-530-3385

Content

Cell viability assay -- S2

Copy of ^{1}H and ^{13}C NMR -- S4

Cell viability assay

Human GFs viability after the DPIE derivative treatment was investigated using the EZ-Cytox Cell viability assay kit (water-soluble tetrazolium salt method). The experiment was performed as manufacturer's protocol. Briefly, the derivatives were treated for 24 hours after seeding the cells (1×10^4 cells per well). Then WST reagent solution (10 μl) was added to each well of a 96-well microplate that contained 100 μl of cells in the culture medium. The plate was then incubated for 1 h at 37 °C. The absorbance was measured at 450 nm using a microplate reader. At the same time, culture medium without cells were incubated for 1day to obtain the background signal. We calculated the final value: total signal−background signal=original signal, (original signal/control signal)×100=Survival (%).

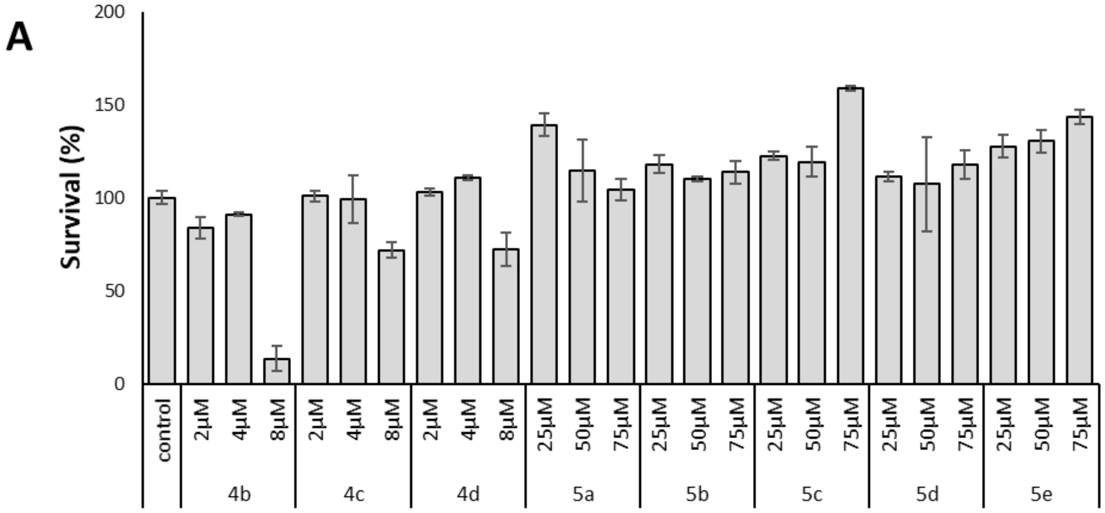

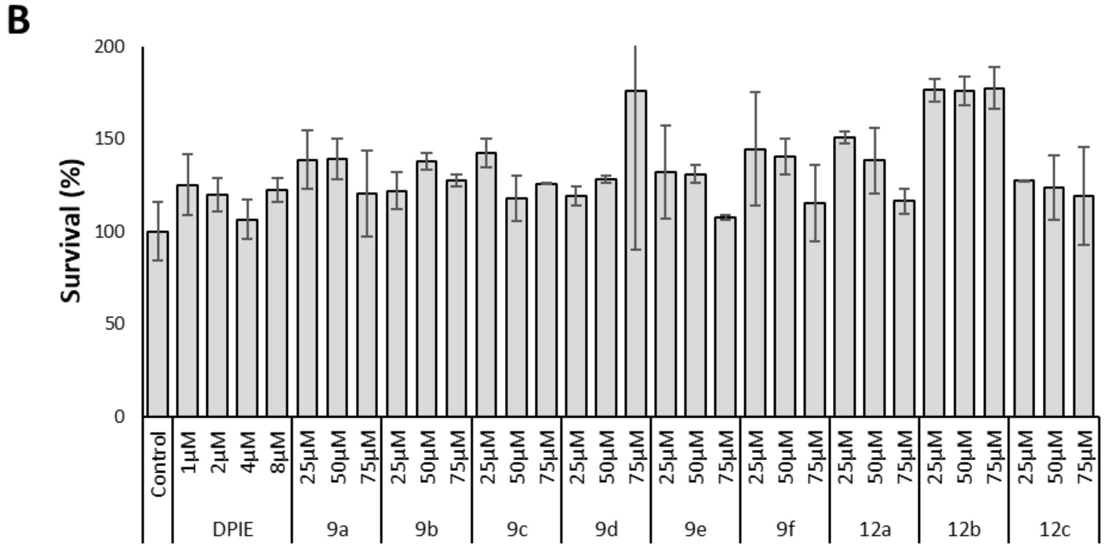

Figure S1 The viability test was performed using water-soluble tetrazolium salt (WST) method on human GFs. The cells were incubated with DPIE derivatives for 24 hours were at indicated concentration. Group 4 chemicals (A) were tested at 2-8 µM, DPIE (B) was tested at 1 - 8 µM, and group 5(A), 9(B), 12(B) chemicals were tested at 25 - 75 µM. Control indicates hGFs treated with solvent, DMSO. Error bars means standard deviations from duplicated experiment.

1,2-diphenyl-1H-indole(2a)

^{1}H NMR

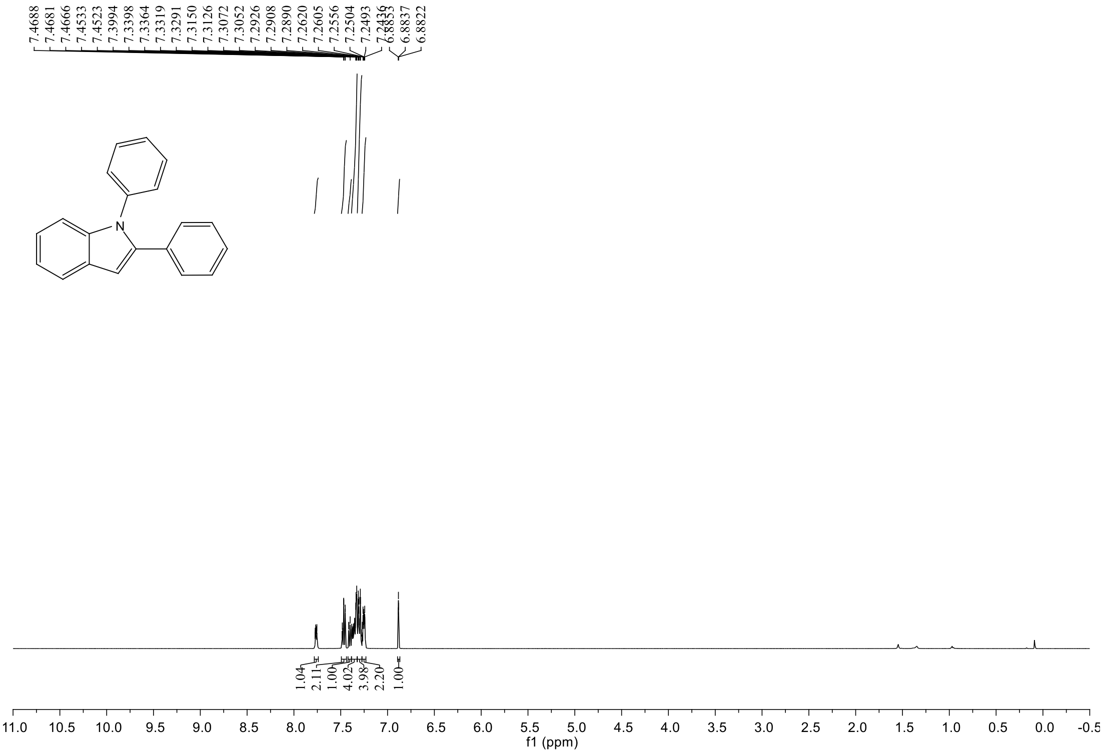

1,2-diphenyl-1H-indole(2a)

^{13}C-NMR

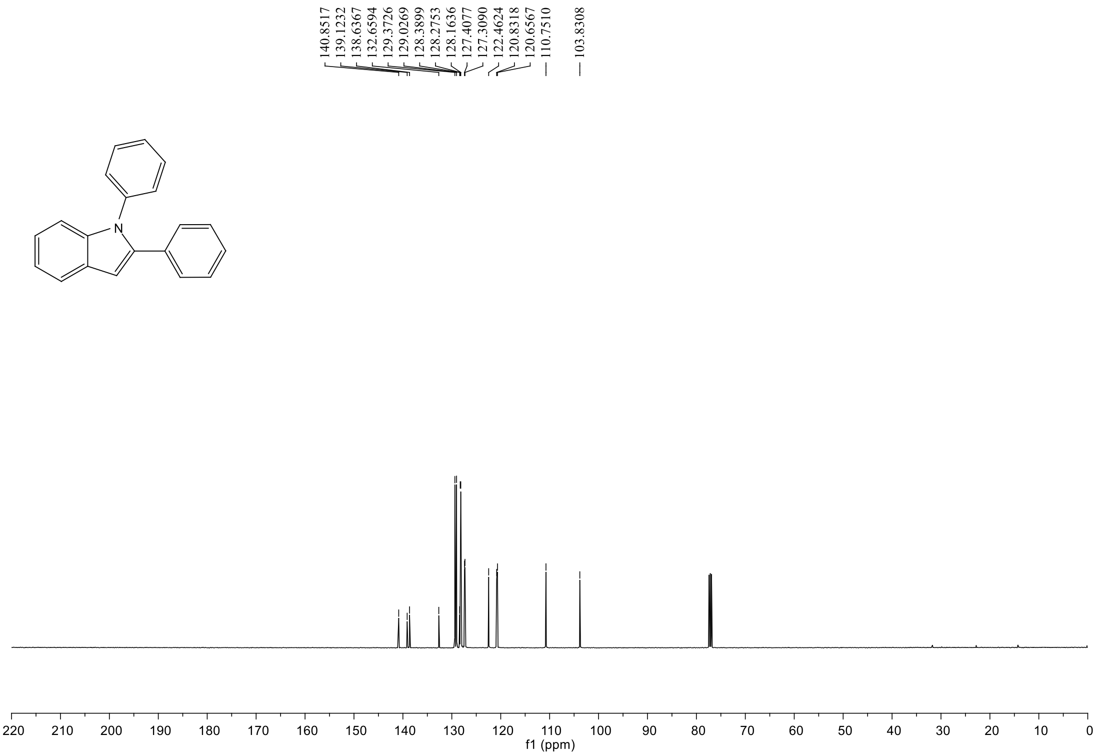

1-([1,1'-biphenyl]-4-yl)-2-phenyl-1H-indole(2b)

^{1}H NMR

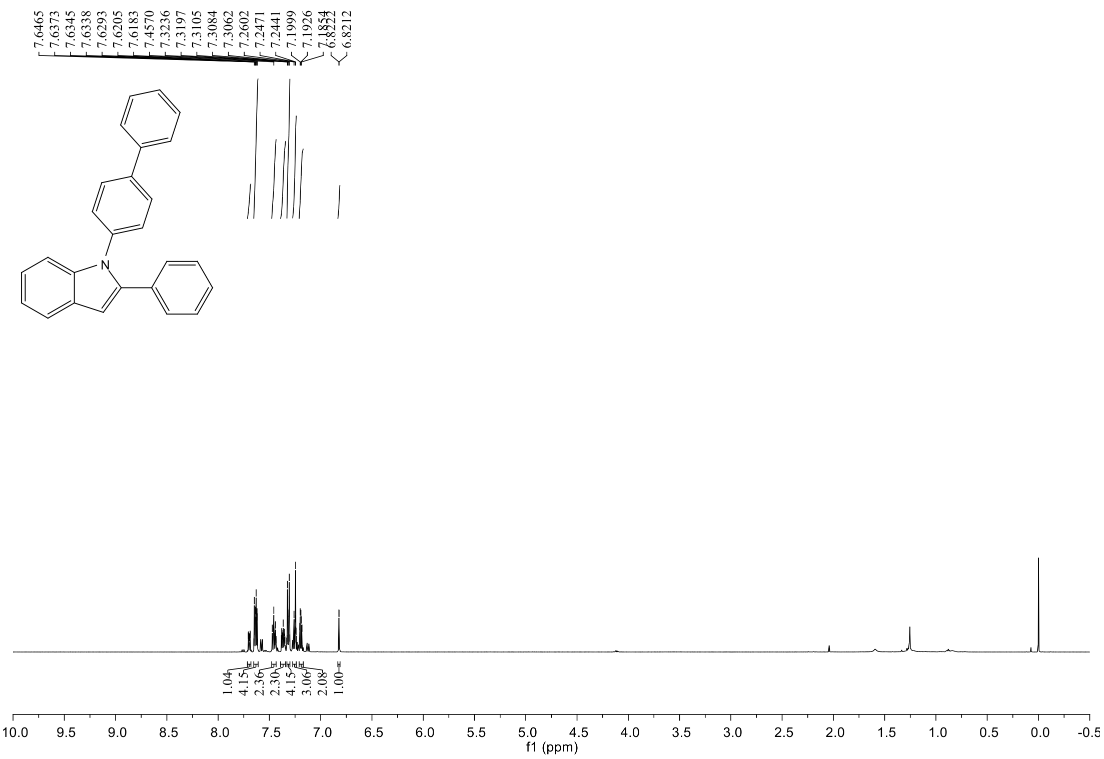

1-([1,1'-biphenyl]-4-yl)-2-phenyl-1H-indole(2b)

^{13}C-NMR

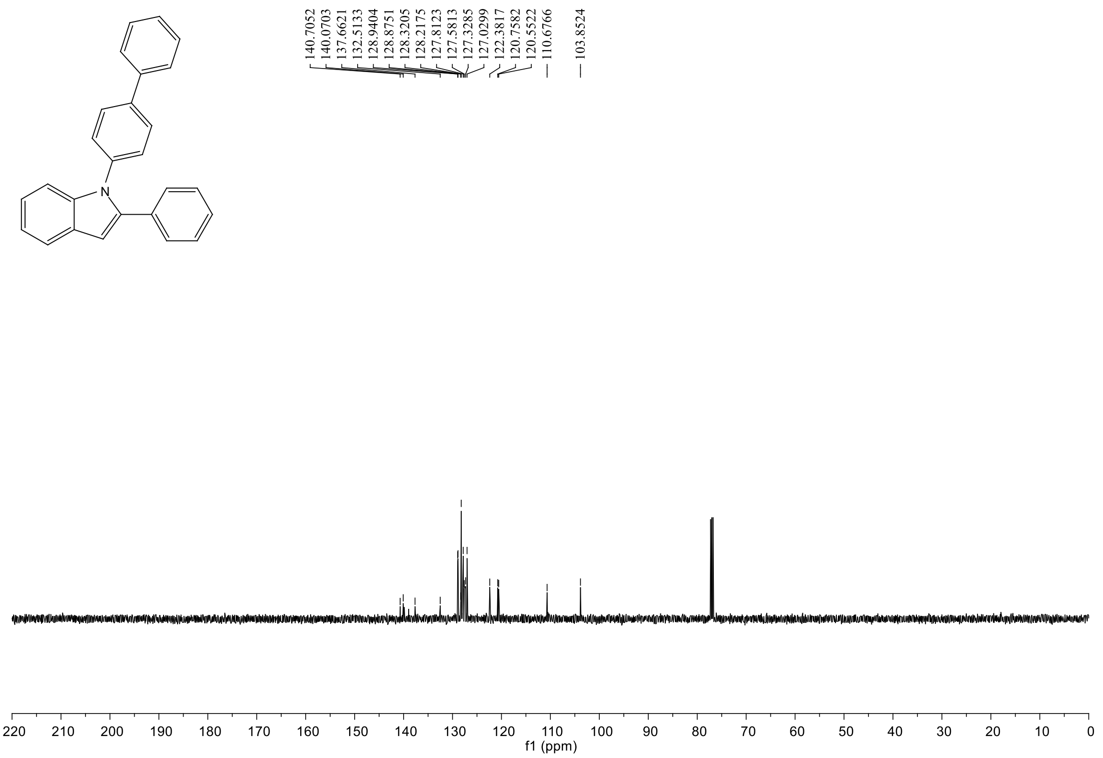

2-phenyl-1-(4-(trifluoromethyl)phenyl)-1H-indole(2c)

^{1}H NMR

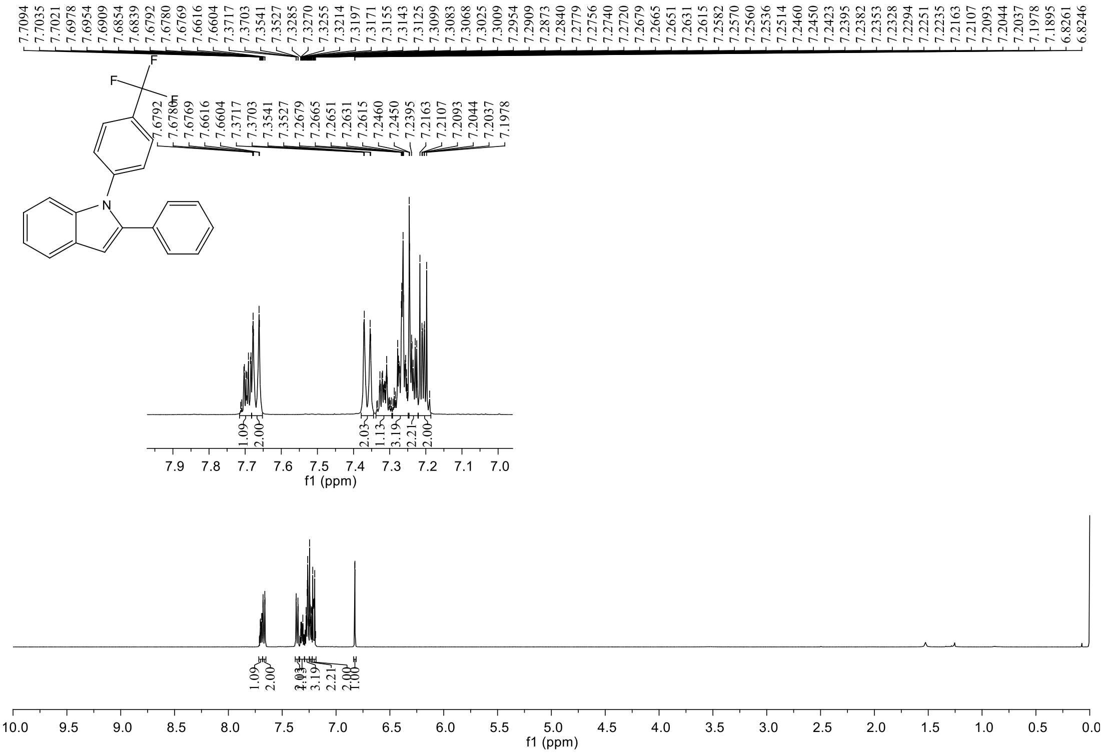

2-phenyl-1-(4-(trifluoromethyl)phenyl)-1H-indole(2c)

^{13}C-NMR

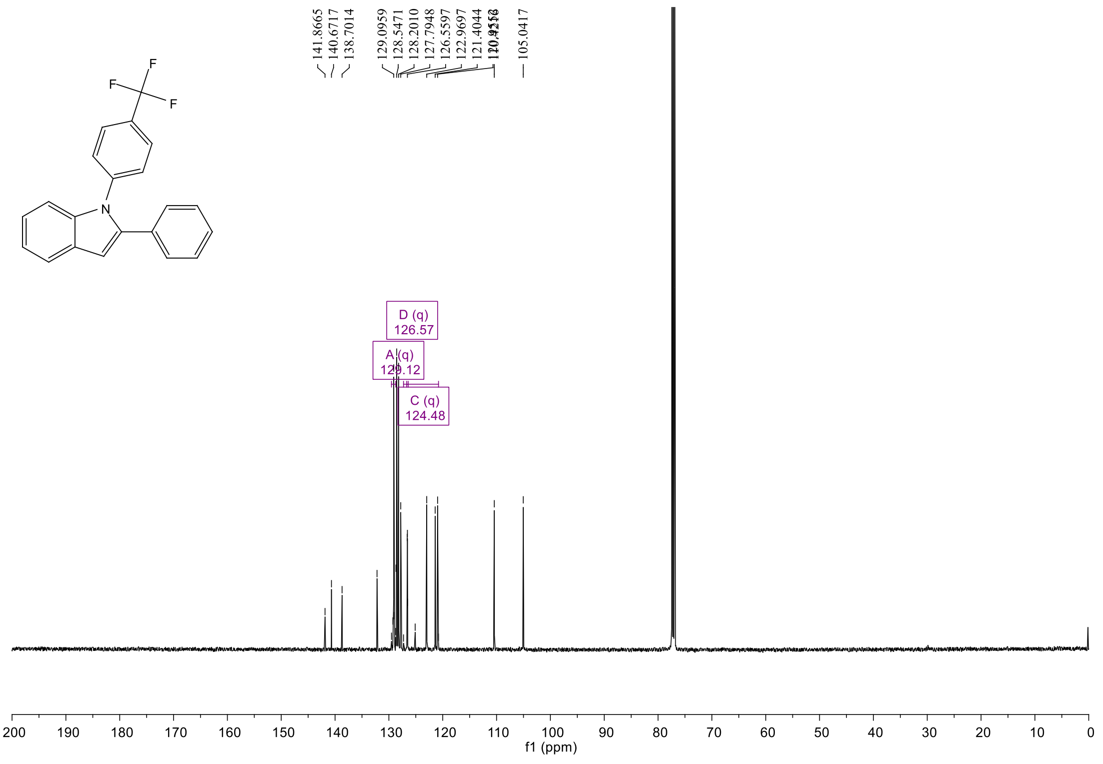

1-(9,9-dimethyl-9H-fluoren-2-yl)-2-phenyl-1H-indole(2d)

^{1}H NMR

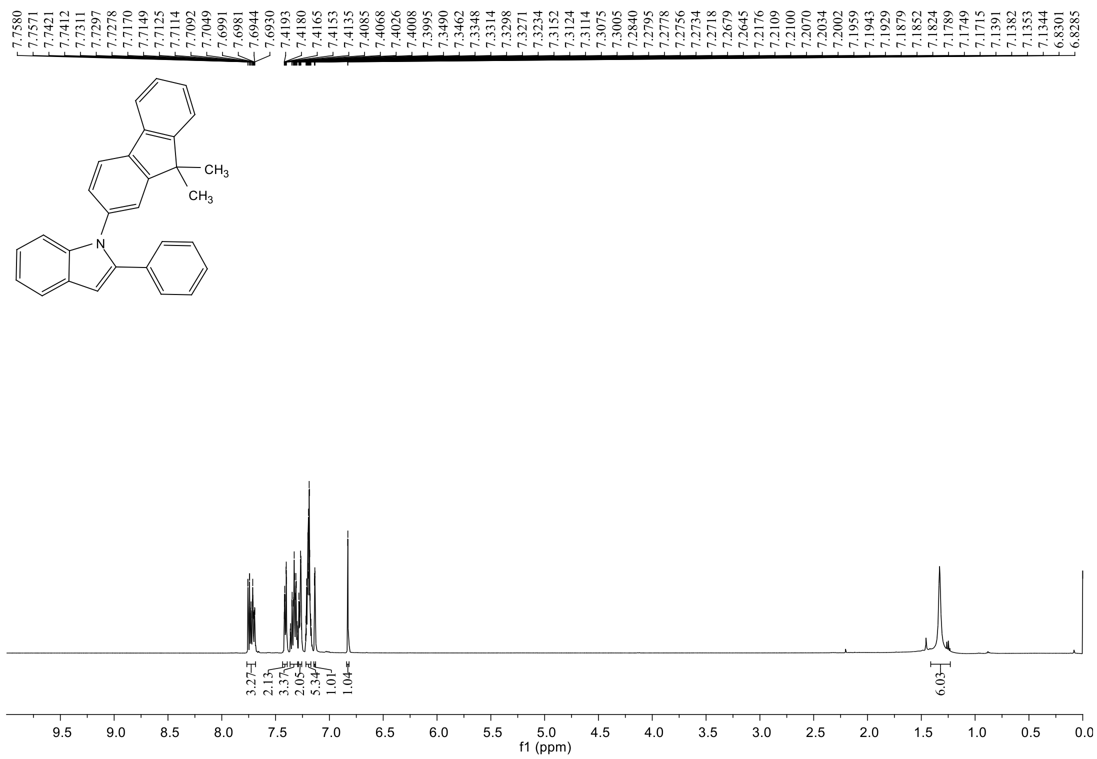

1-(9,9-dimethyl-9H-fluoren-2-yl)-2-phenyl-1H-indole(2d)

^{13}C-NMR

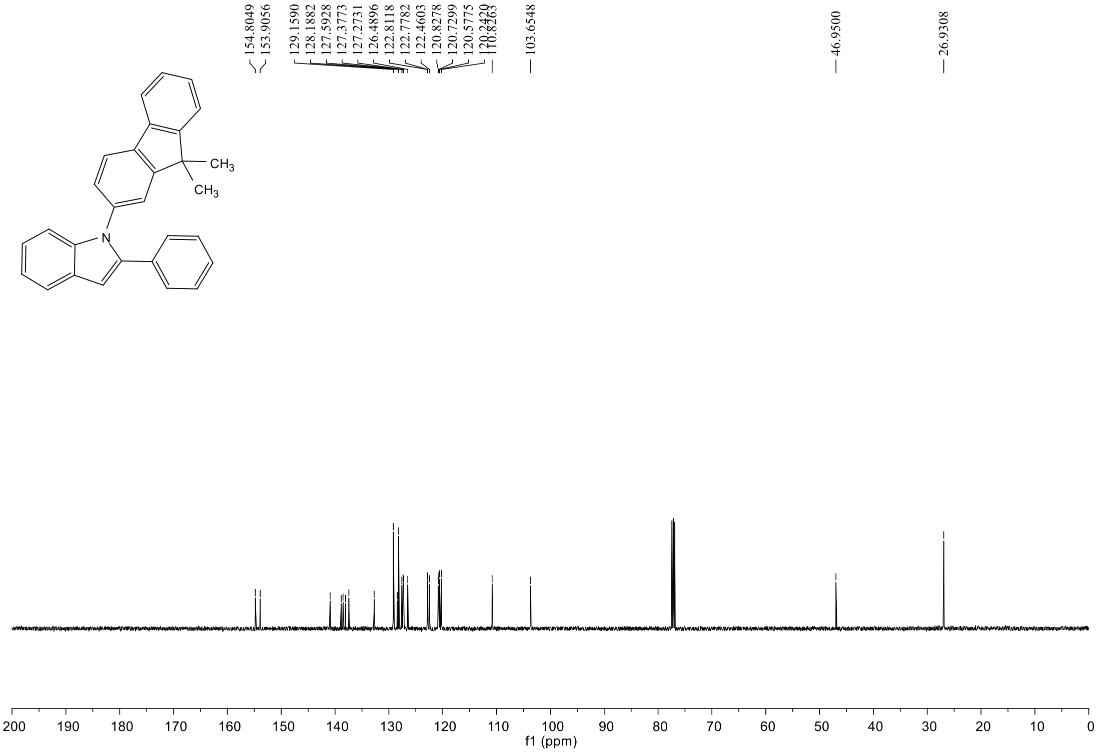

1,2-diphenyl-1H-indole-3-carbaldehyde(3a)

^{1}H NMR

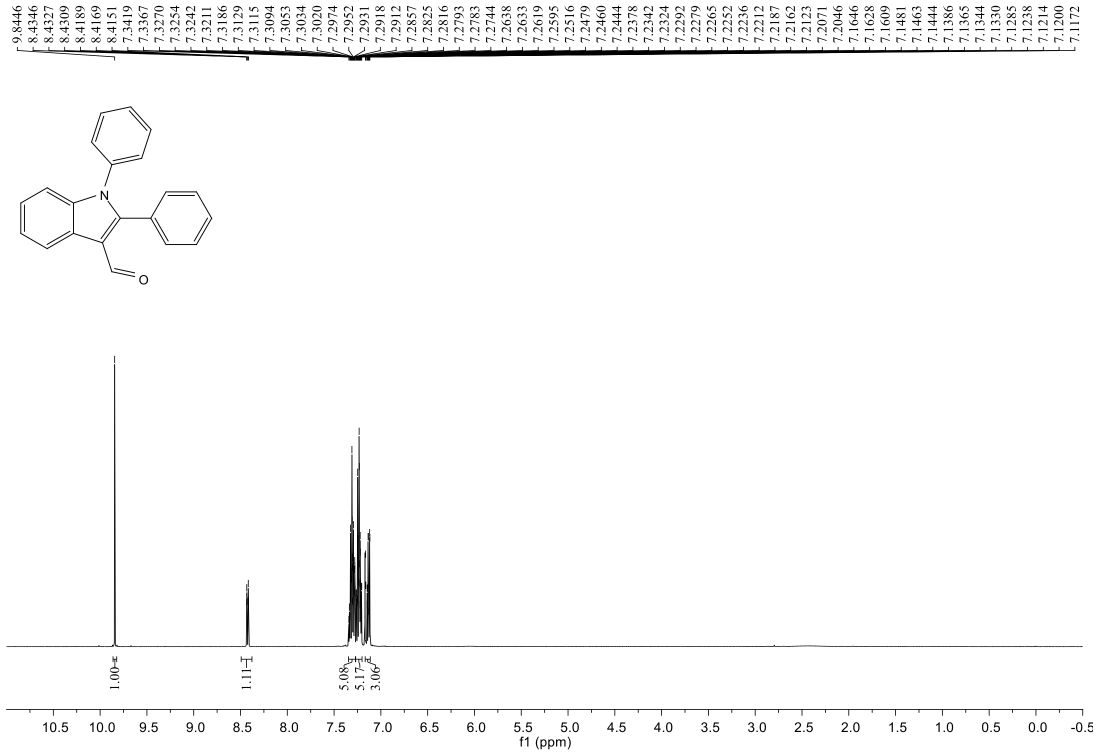

1,2-diphenyl-1H-indole-3-carbaldehyde(3a)

^{13}C-NMR

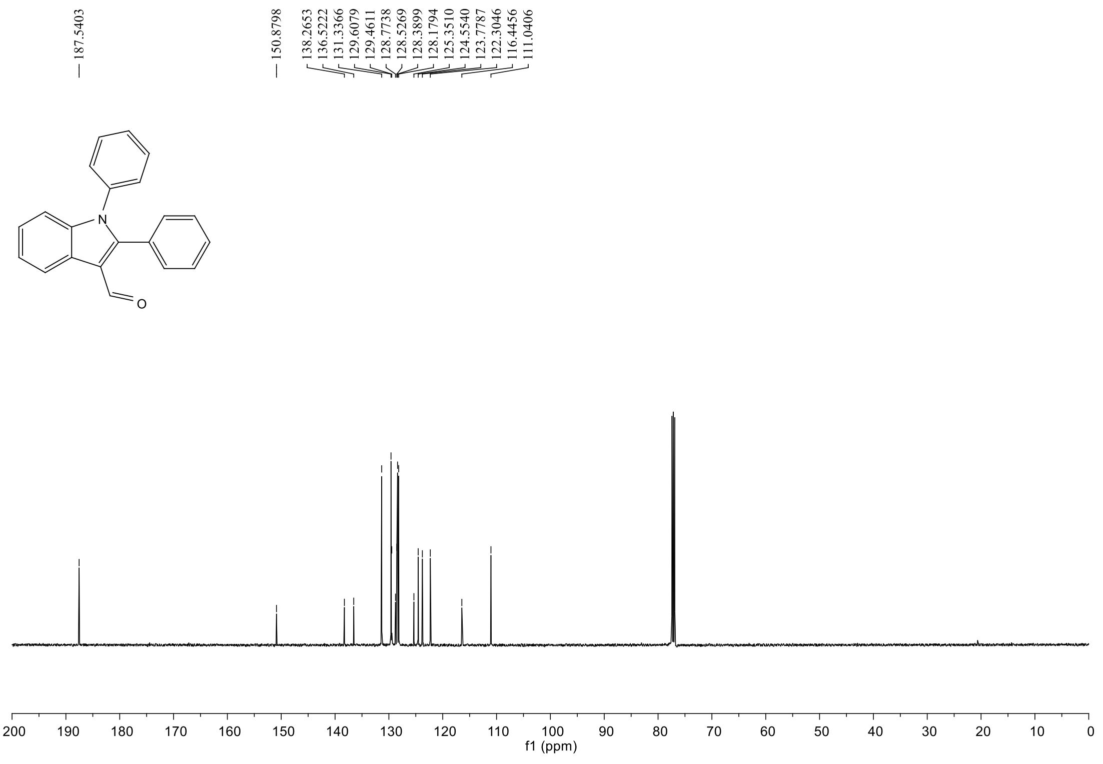

1-([1,1'-biphenyl]-4-yl)-2-phenyl-1H-indole-3-carbaldehyde(3b)

^{1}H NMR

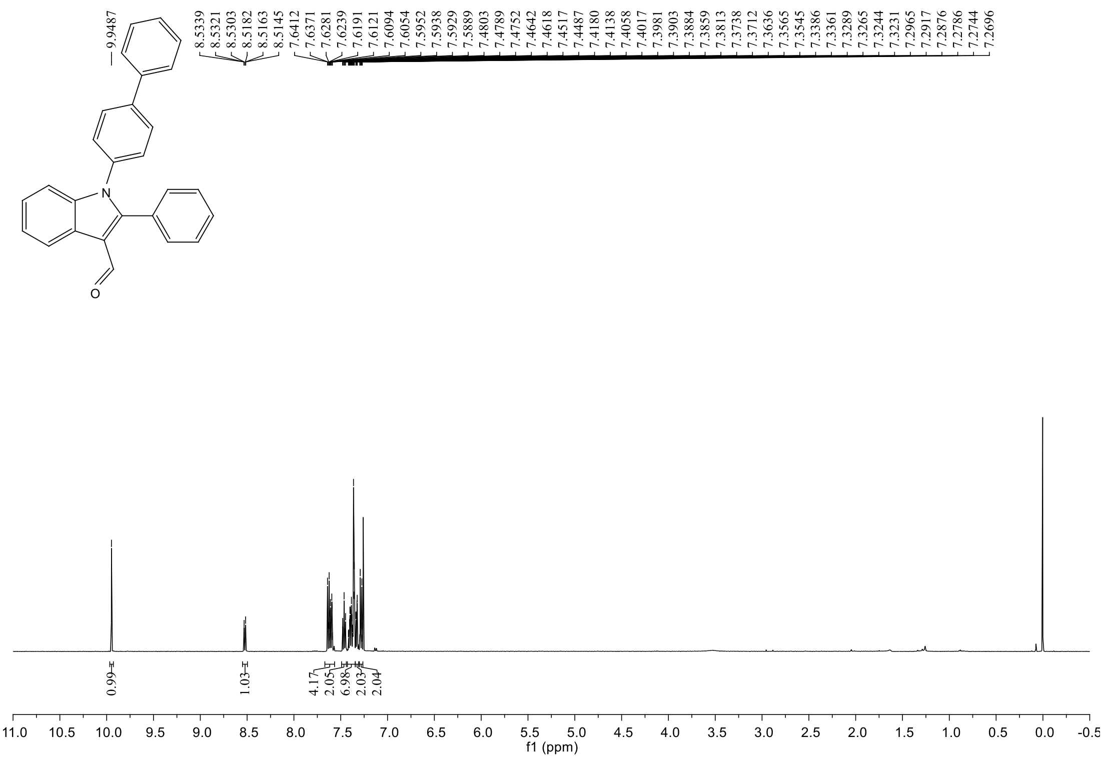

1-([1,1'-biphenyl]-4-yl)-2-phenyl-1H-indole-3-carbaldehyde(3b)

^{13}C-NMR

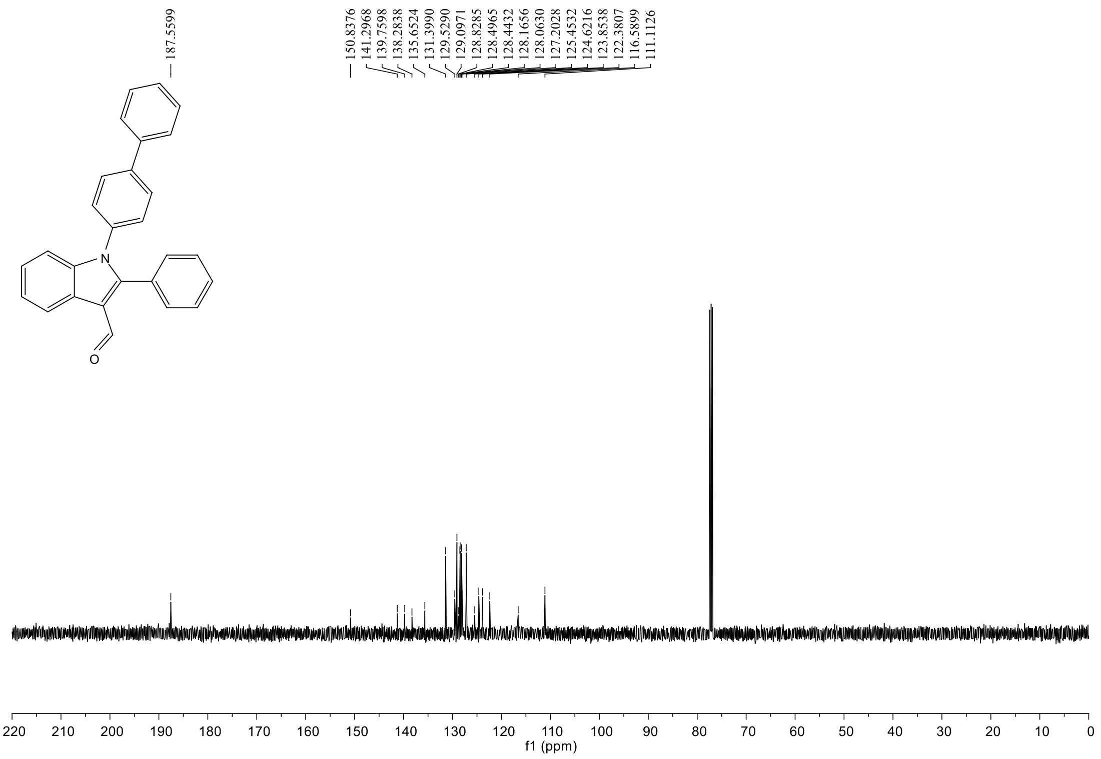

2-phenyl-1-(4-(trifluoromethyl)phenyl)-1H-indole-3-carbaldehyde (3c)

^{1}H NMR

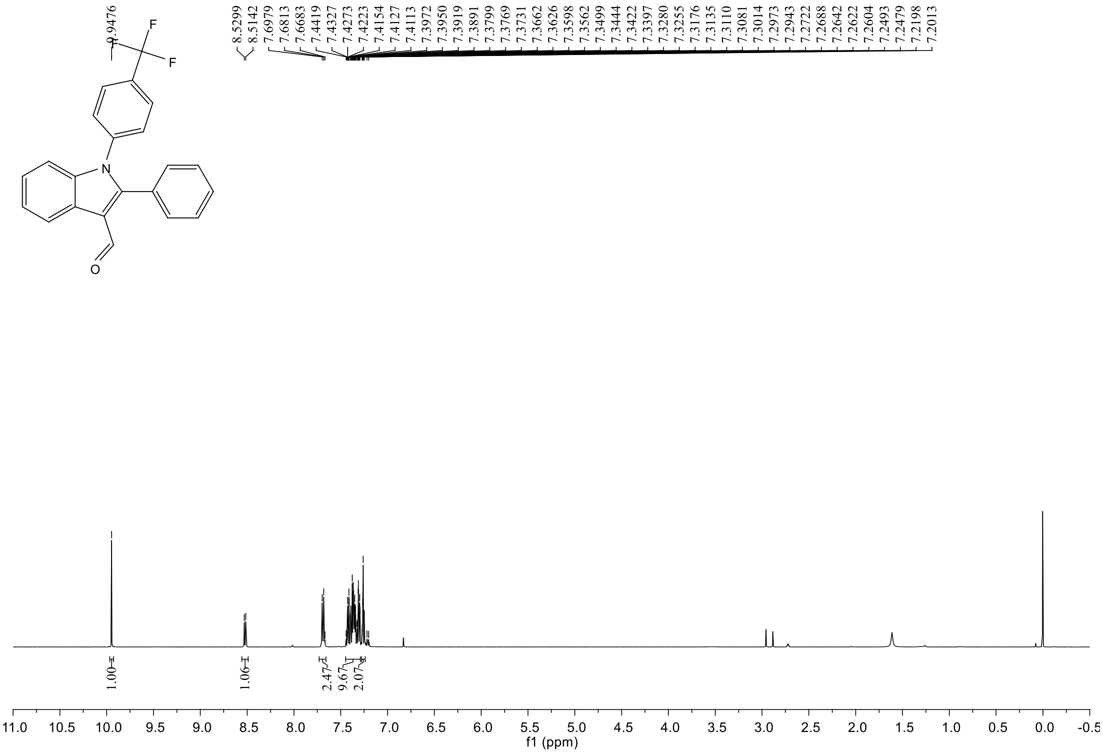

2-phenyl-1-(4-(trifluoromethyl)phenyl)-1H-indole-3-carbaldehyde (3c)

^{13}C-NMR

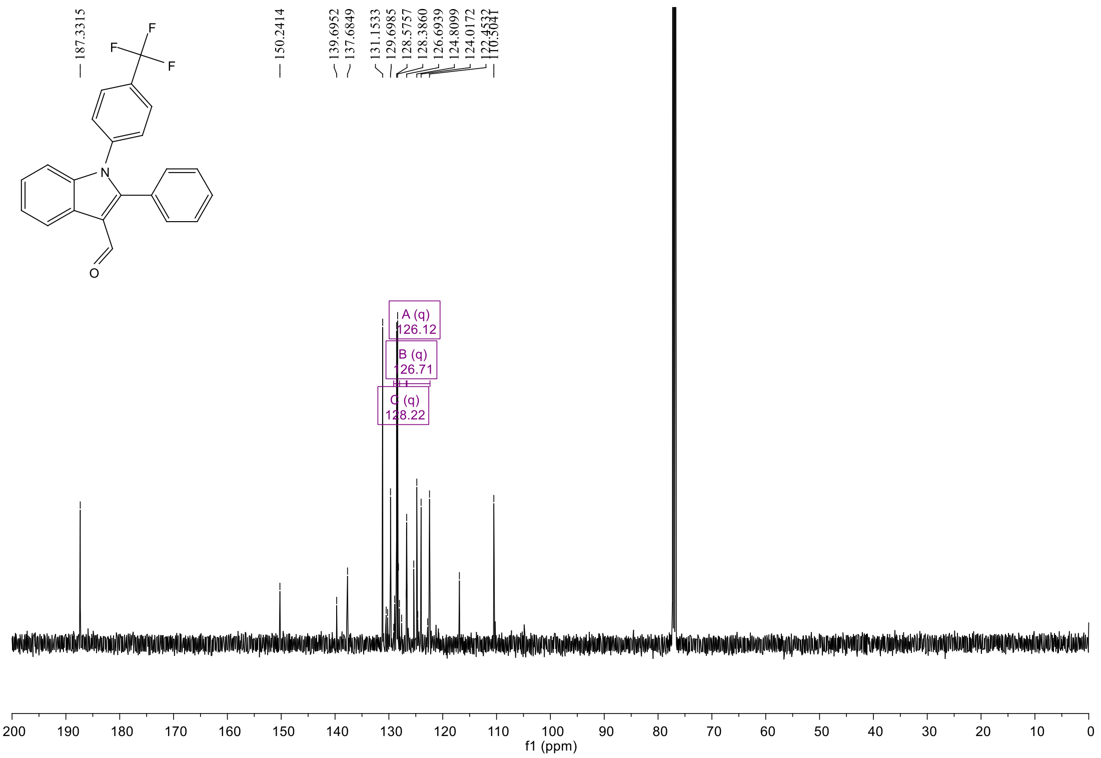

1-(9,9-dimethyl-9H-fluoren-2-yl)-2-phenyl-1H-indole-3-carbaldehyde(3d)

^{1}H NMR

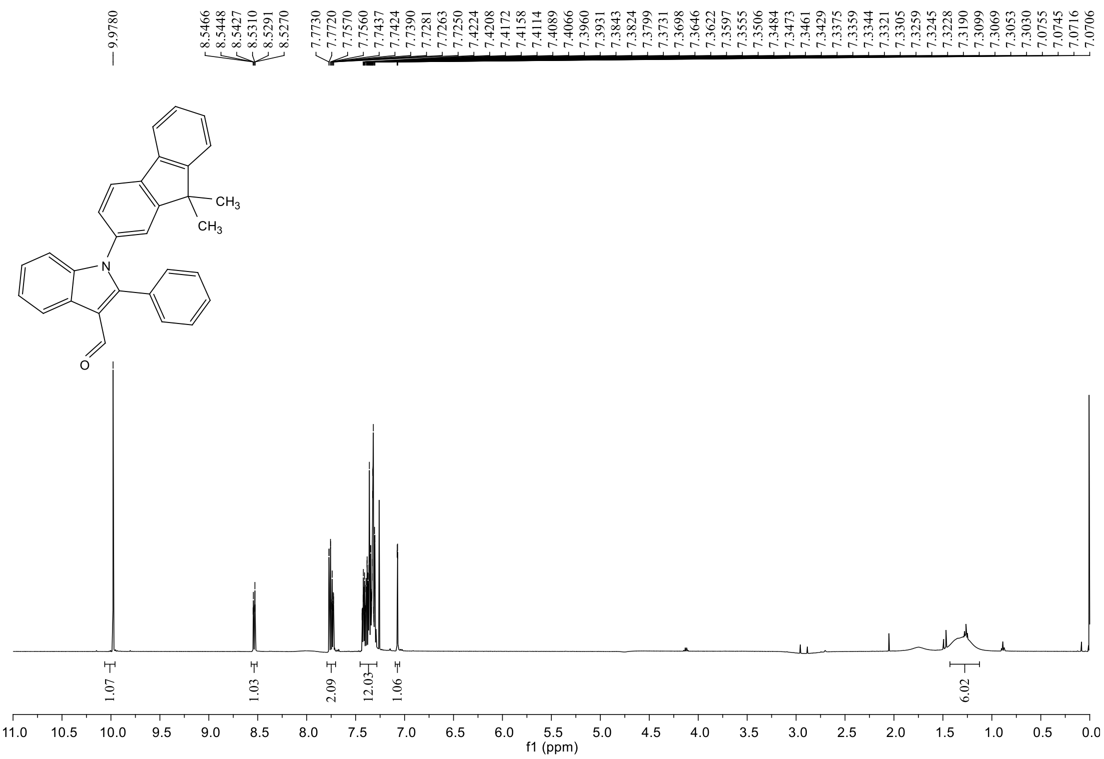

1-(9,9-dimethyl-9H-fluoren-2-yl)-2-phenyl-1H-indole-3-carbaldehyde(3d)

^{13}C-NMR

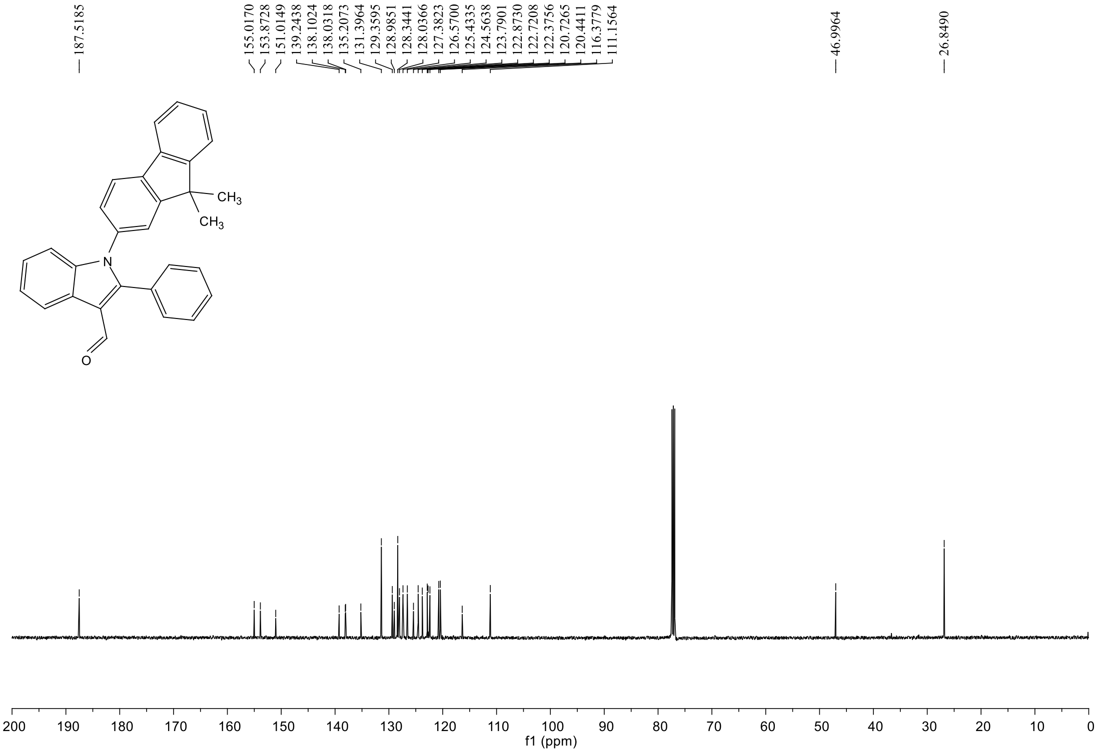

2-(1,2-diphenyl-1H-indol-3-yl)ethanamine (4a: DPIE)

^{1}H NMR

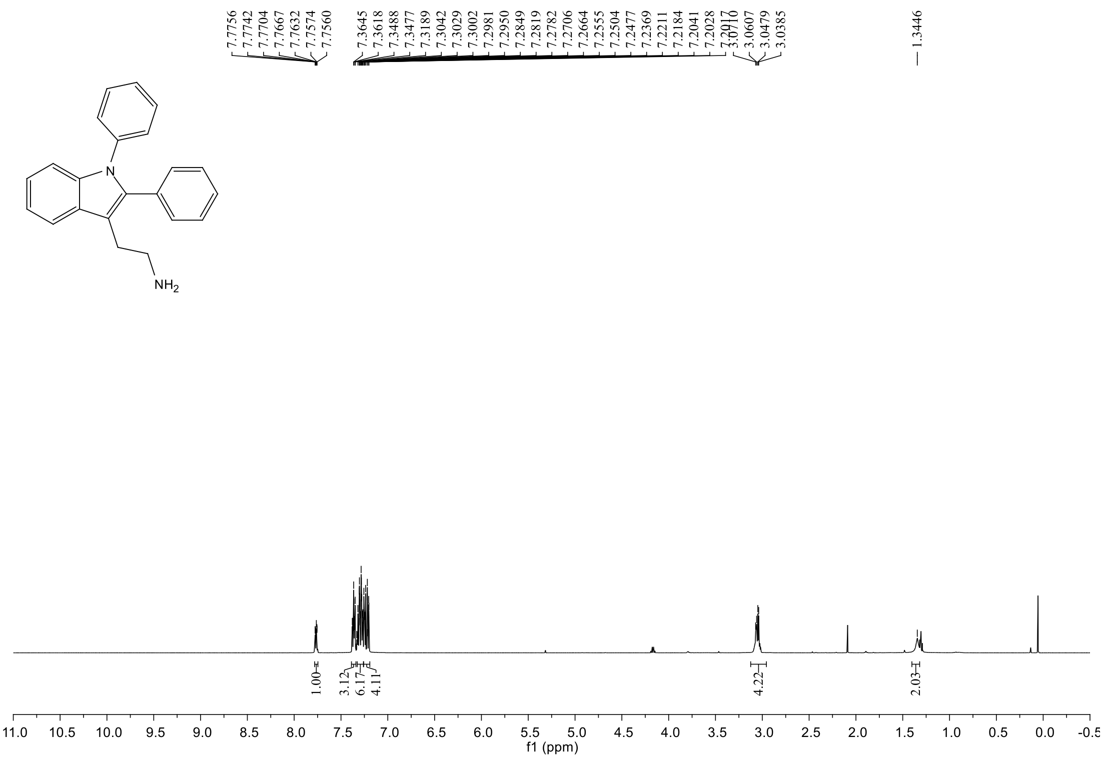

2-(1,2-diphenyl-1H-indol-3-yl)ethanamine (4a: DPIE)

^{13}C-NMR

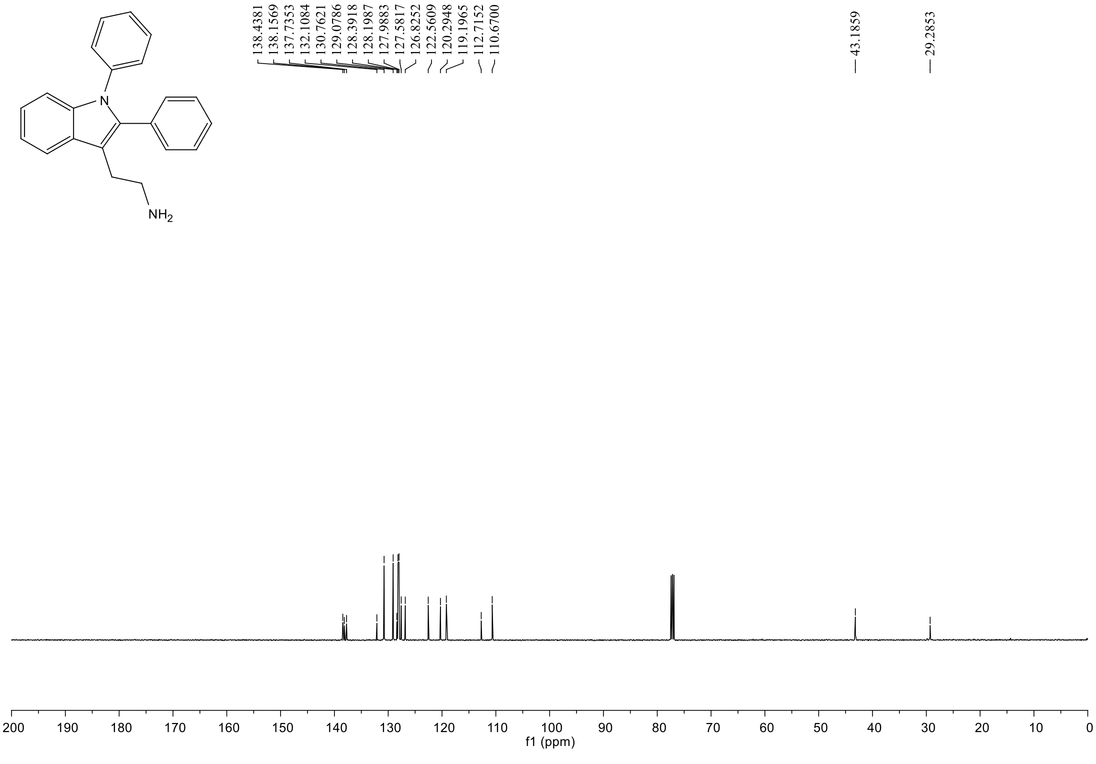

2-(1,2-diphenyl-1H-indol-3-yl)ethanamine (4b)

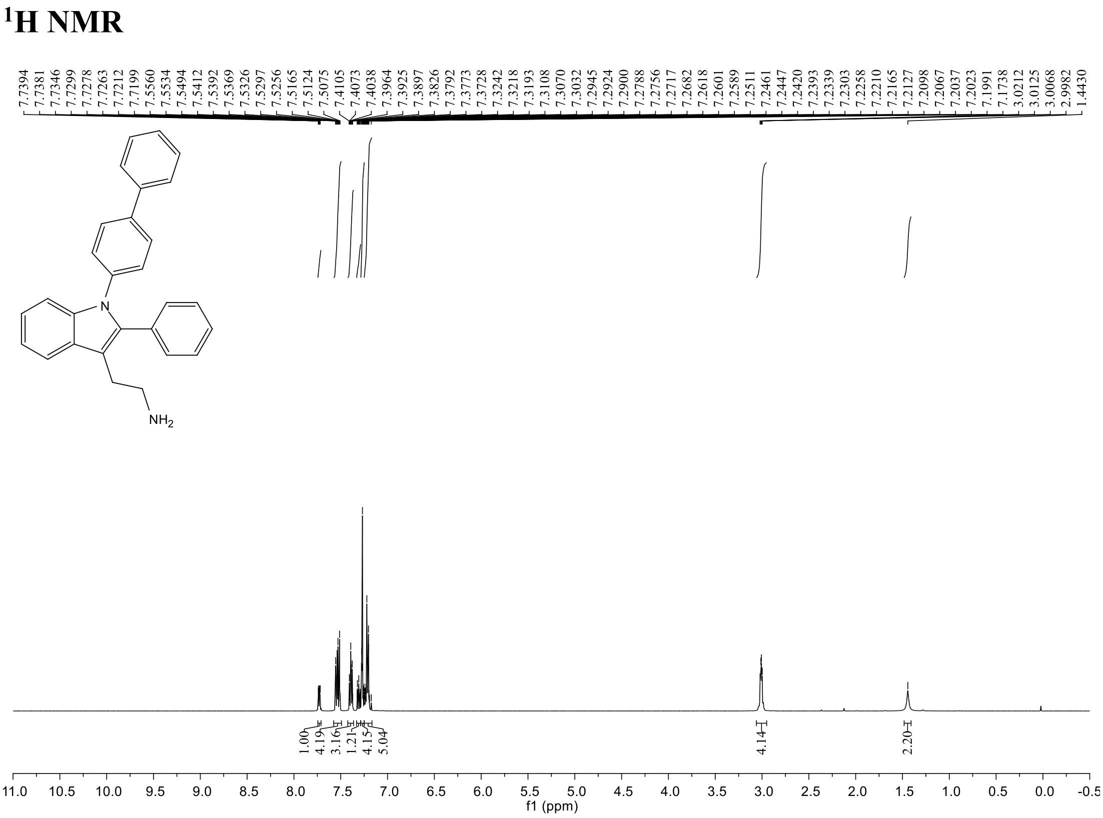

2-(1,2-diphenyl-1H-indol-3-yl)ethanamine (4b)

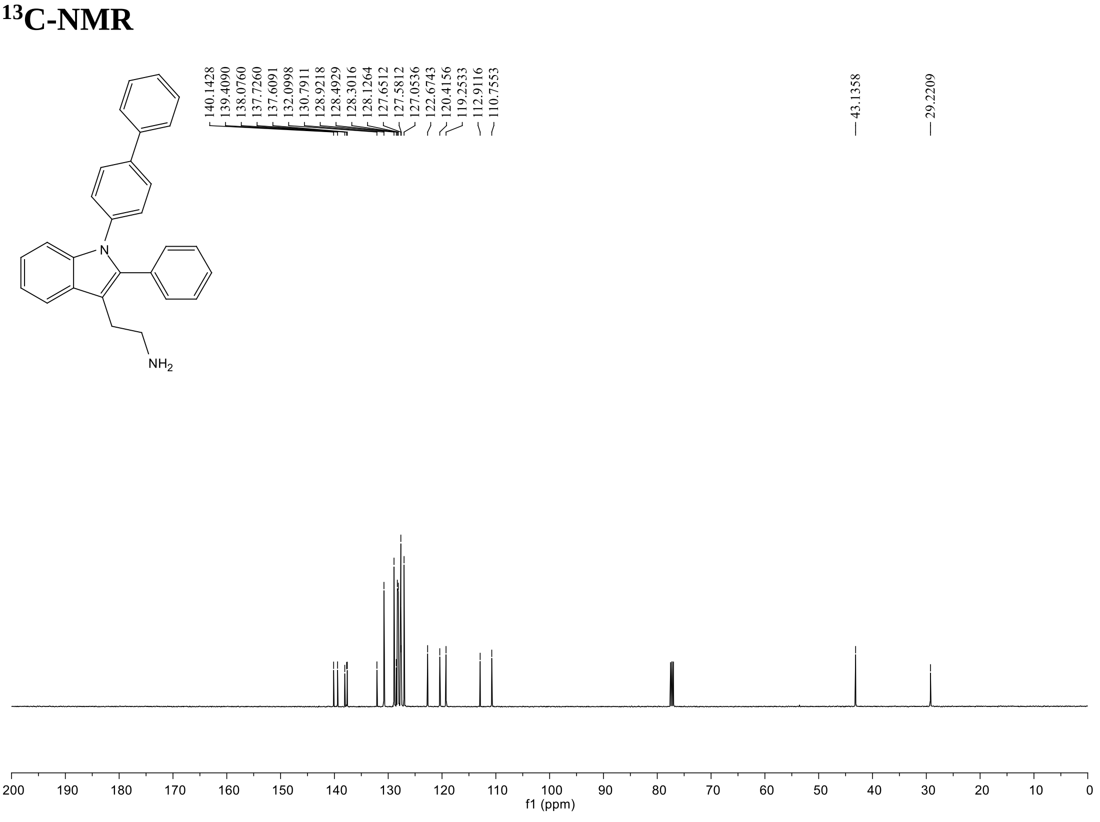

2-(2-phenyl-1-(4-(trifluoromethyl)phenyl)-1H-indol-3-yl)ethanamine(4c)

^{1}H NMR

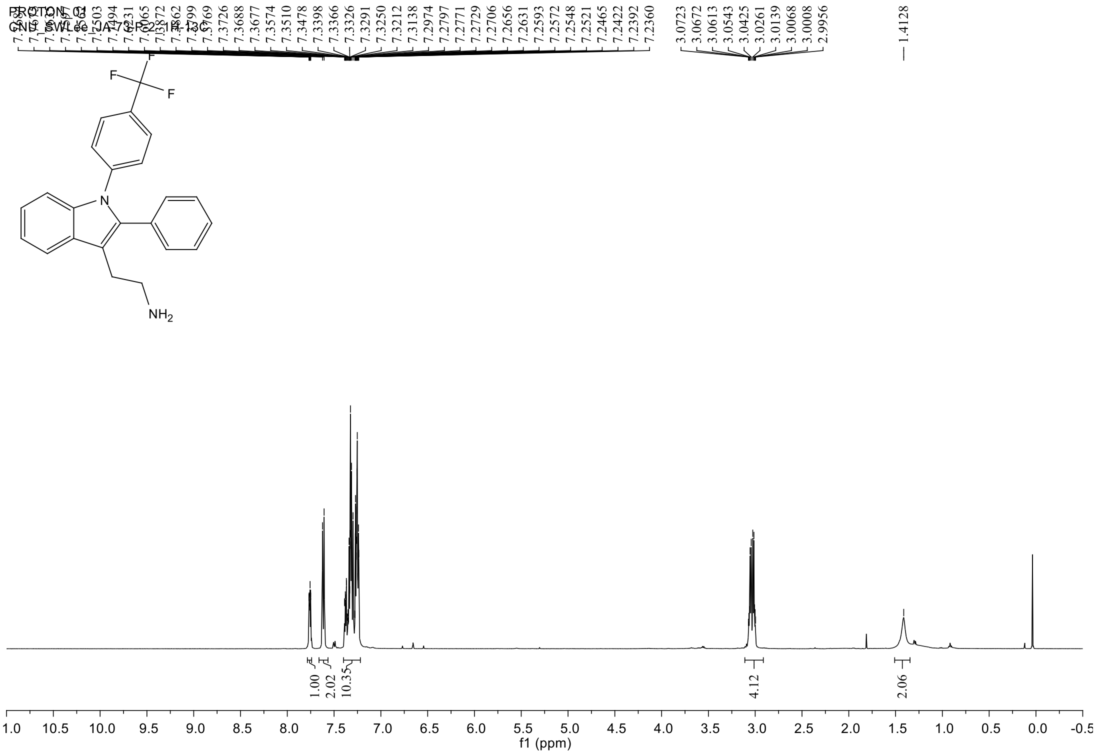

2-(2-phenyl-1-(4-(trifluoromethyl)phenyl)-1H-indol-3-yl)ethanamine(4c)

^{13}C-NMR

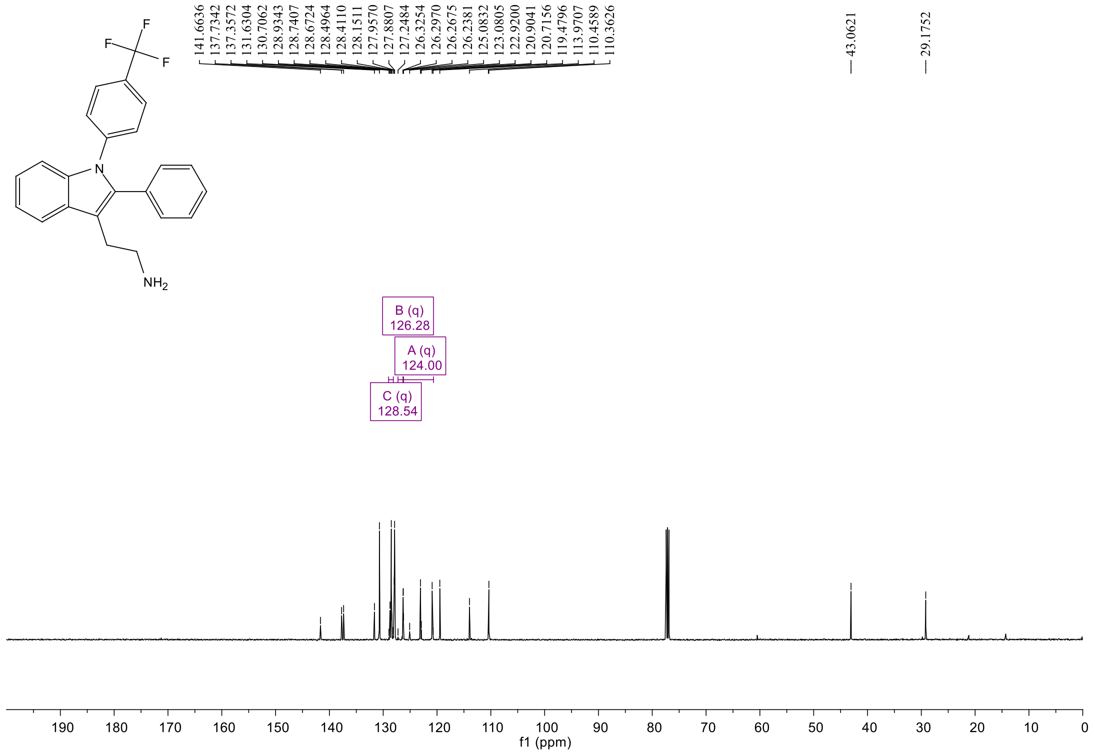

2-(1-(9,9-dimethyl-9H-fluoren-2-yl)-2-phenyl-1H-indol-3-yl)ethanamine (4d)

^{1}H NMR

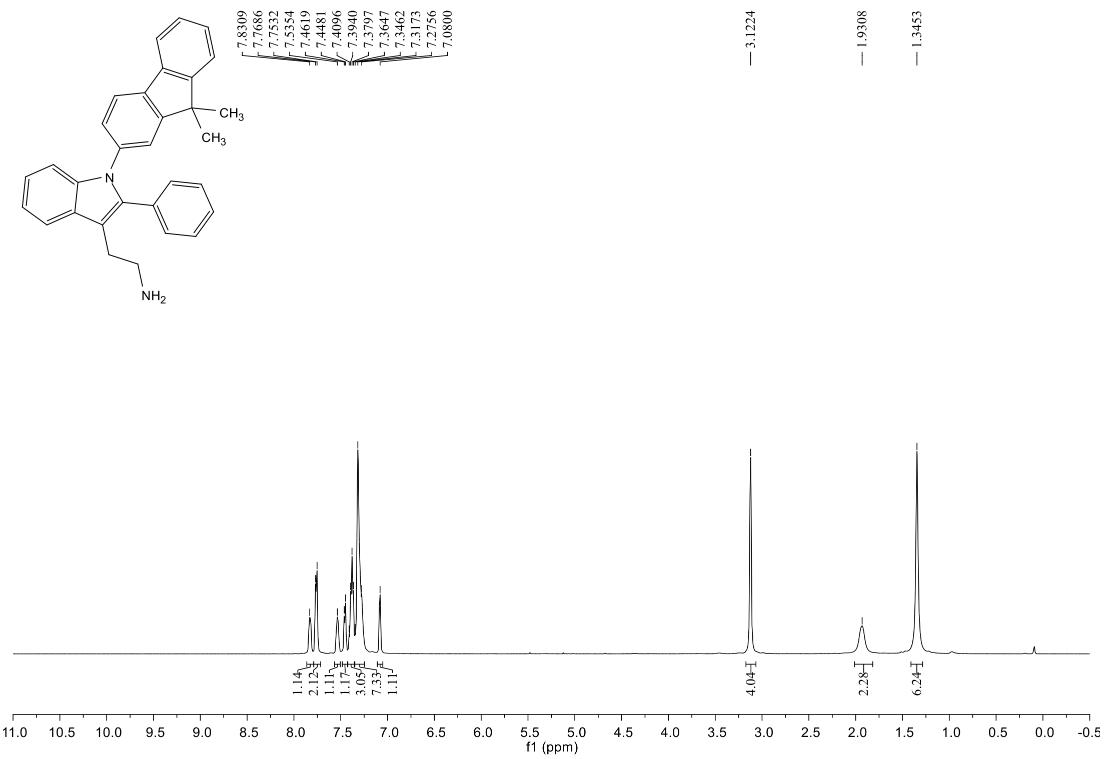

2-(1-(9,9-dimethyl-9H-fluoren-2-yl)-2-phenyl-1H-indol-3-yl)ethanamine (4d)

^{13}C-NMR

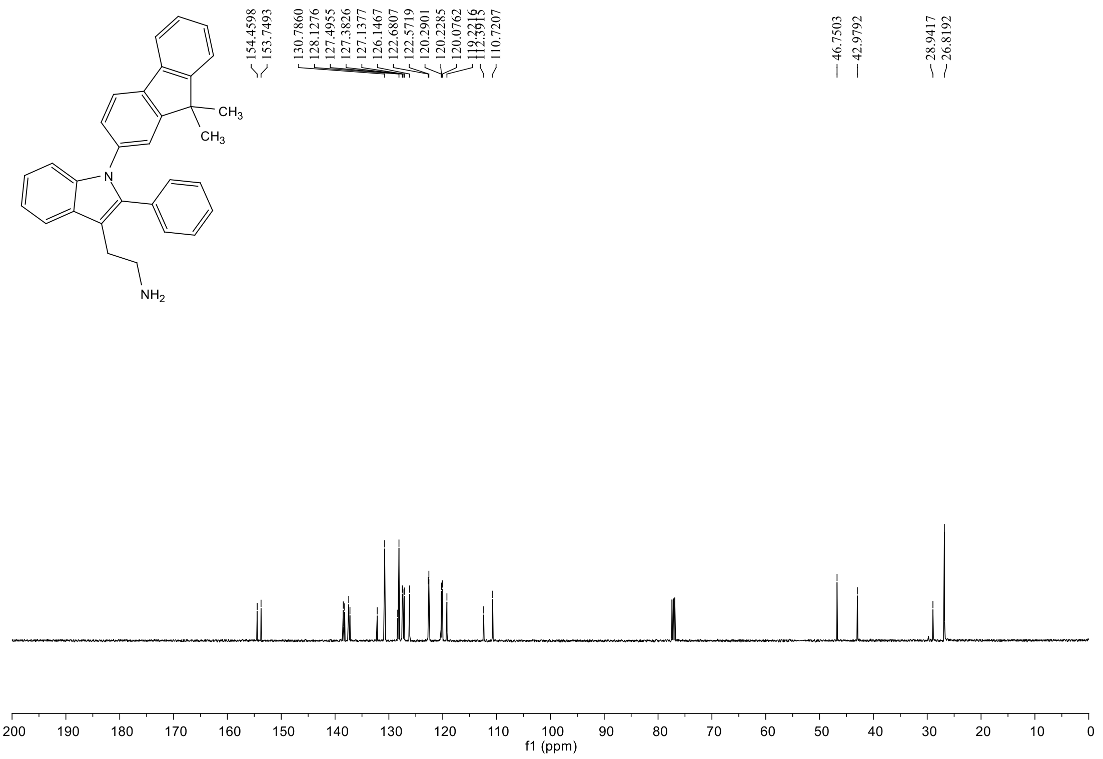

N-((1,2-diphenyl-1H-indol-3-yl)methyl)cyclopropanamine (5a)

^{1}H NMR

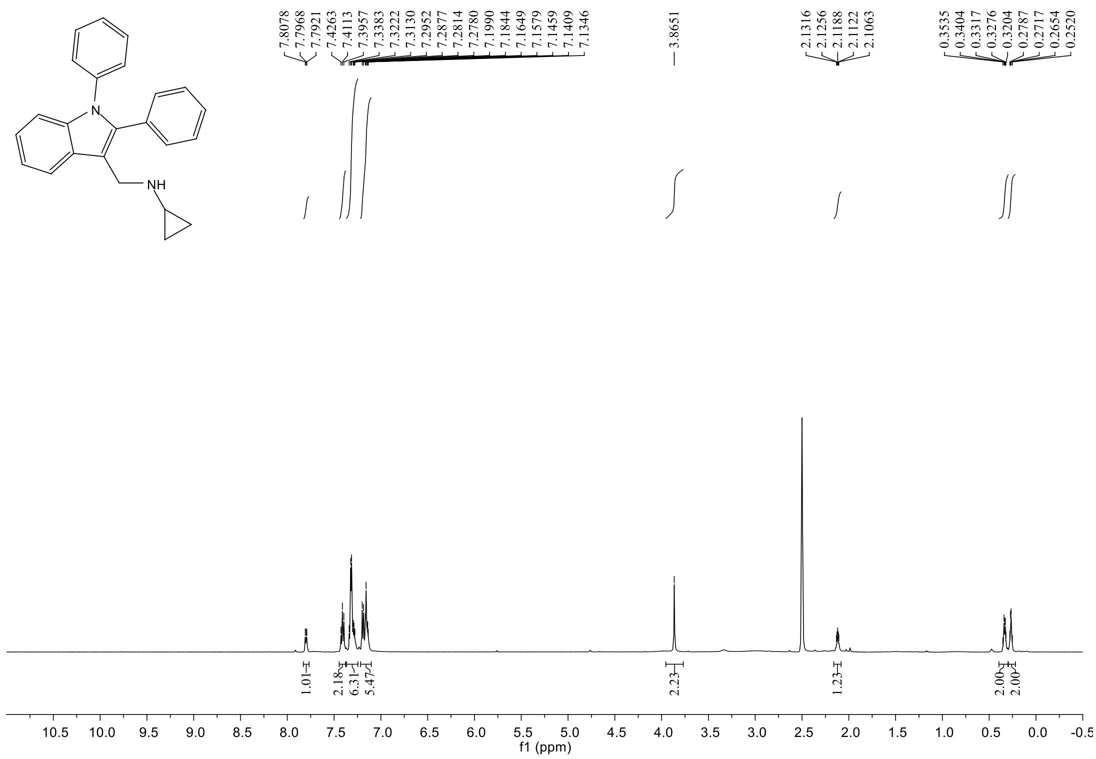

N-((1,2-diphenyl-1H-indol-3-yl)methyl)cyclopropanamine (5a)

^{13}C-NMR

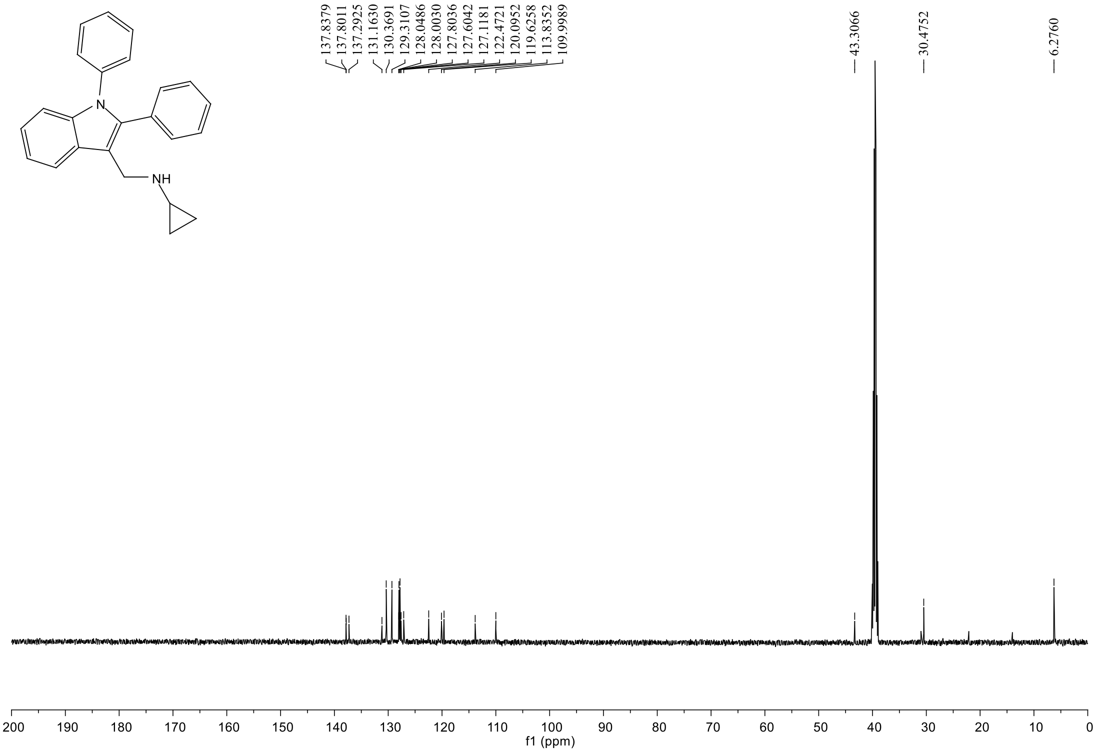

N-((1,2-diphenyl-1H-indol-3-yl)methyl)aniline (5b)

^{1}H NMR

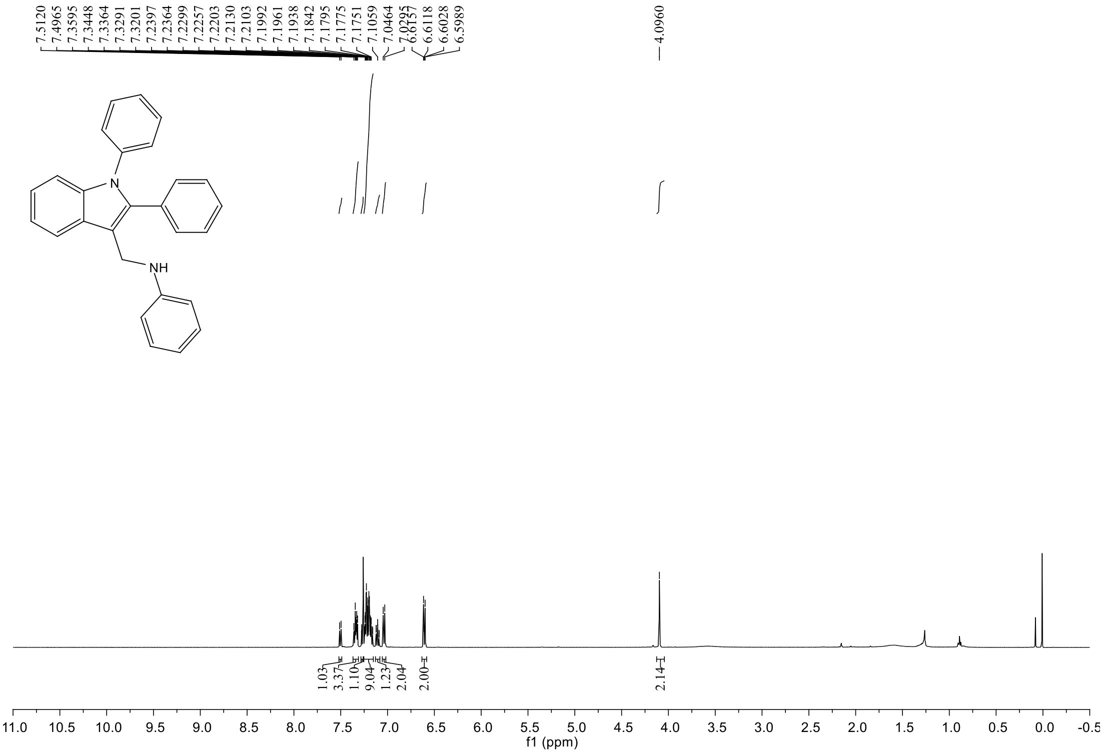

N-((1,2-diphenyl-1H-indol-3-yl)methyl)aniline (5b)

^{13}C-NMR

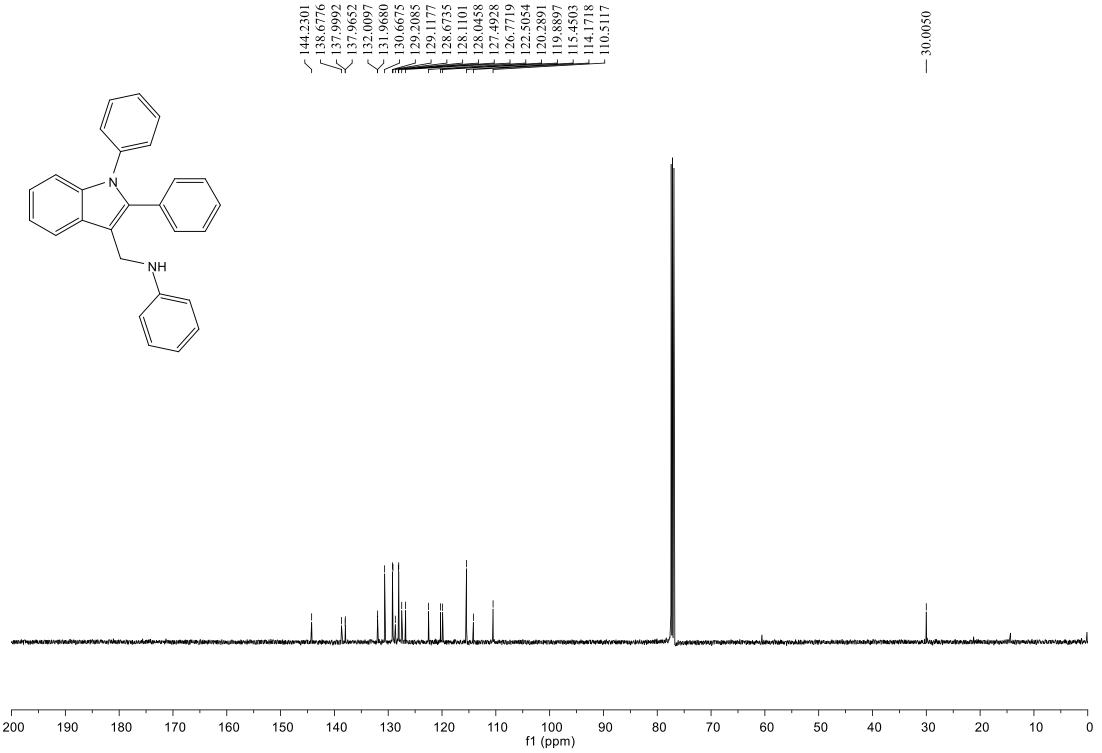

N-((1,2-diphenyl-1H-indol-3-yl)methyl)naphthalen-1-amine (5c)

^{1}H NMR

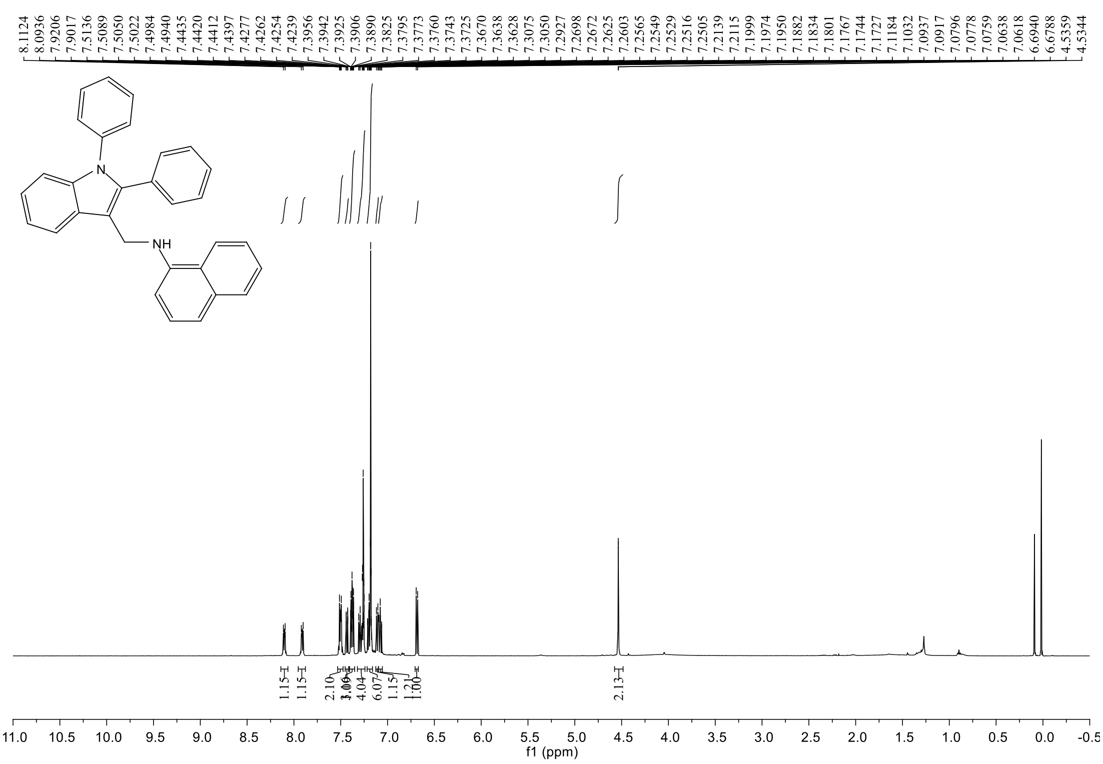

N-((1,2-diphenyl-1H-indol-3-yl)methyl)naphthalen-1-amine (5c)

^{13}C-NMR

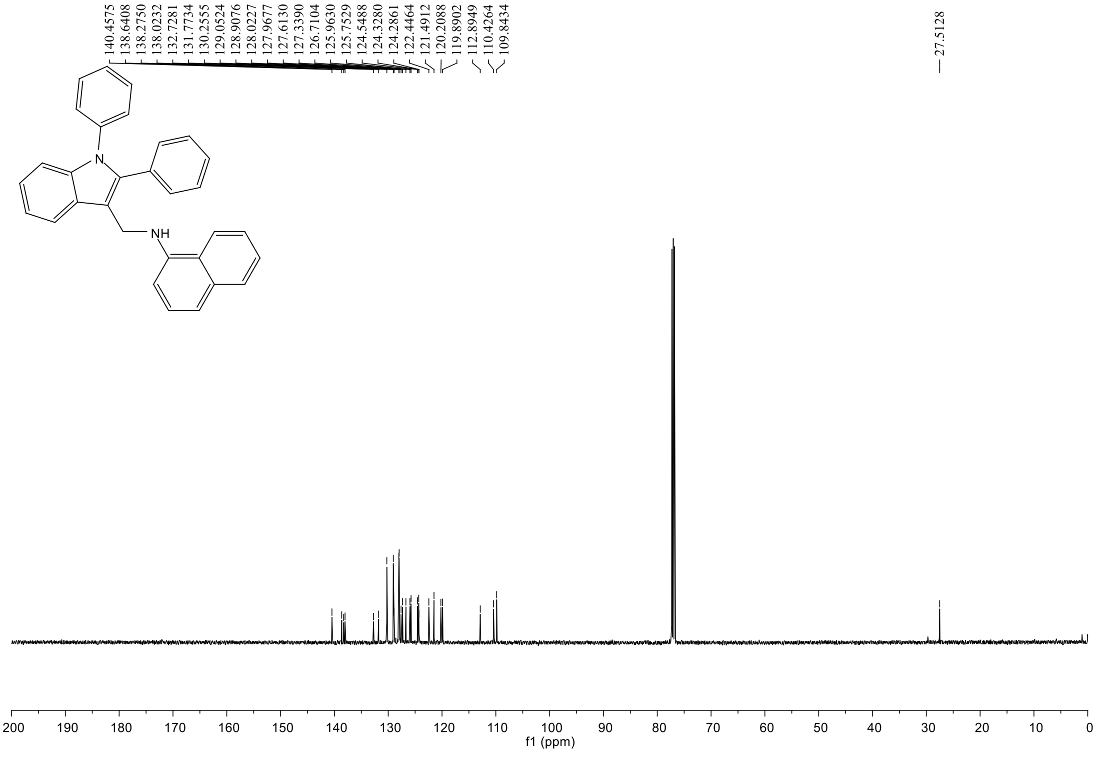

N-((1,2-diphenyl-1H-indol-3-yl)methyl)pyridin-2-amine(5d)

^{1}H NMR

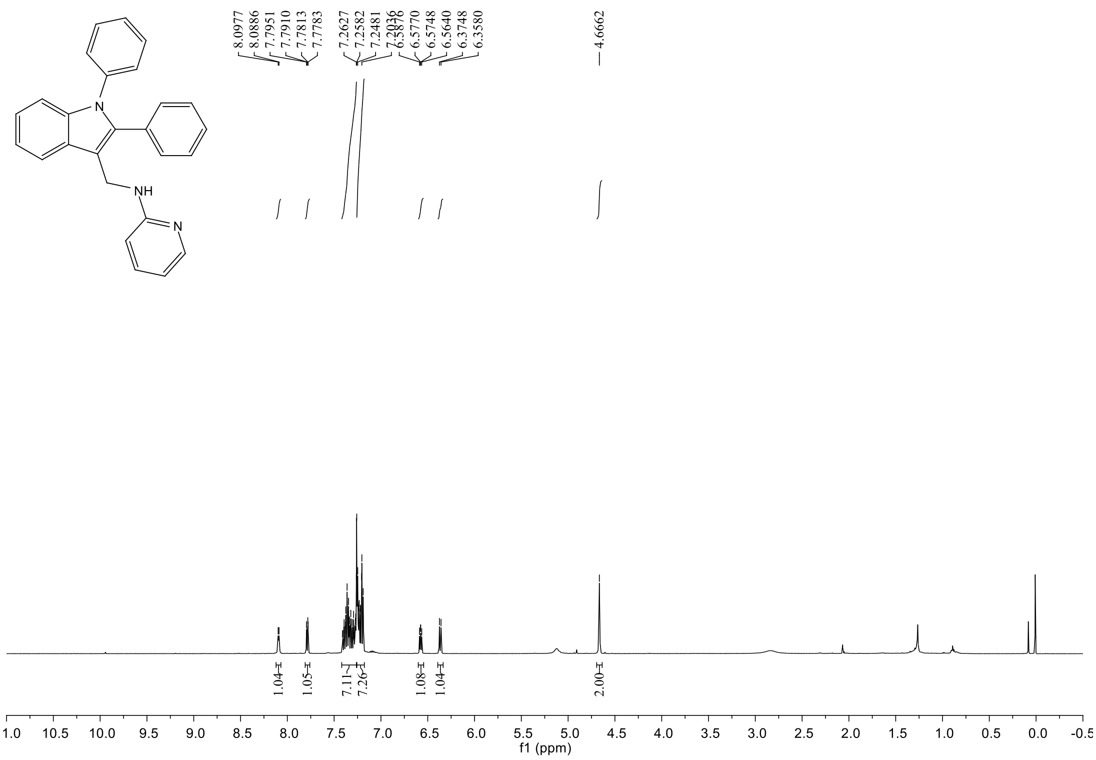

N-((1,2-diphenyl-1H-indol-3-yl)methyl)pyridin-2-amine(5d)

^{13}C-NMR

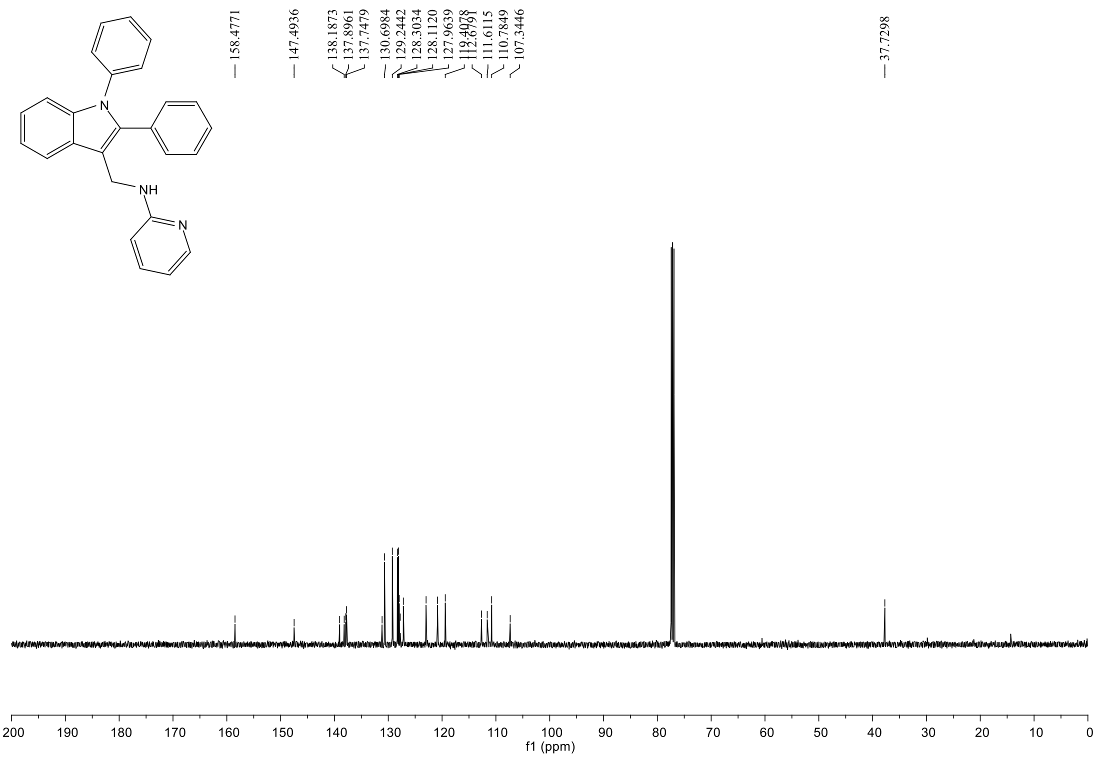

N-((1,2-diphenyl-1H-indol-3-yl)methyl)-N-phenylaniline(5e)

^{1}H NMR

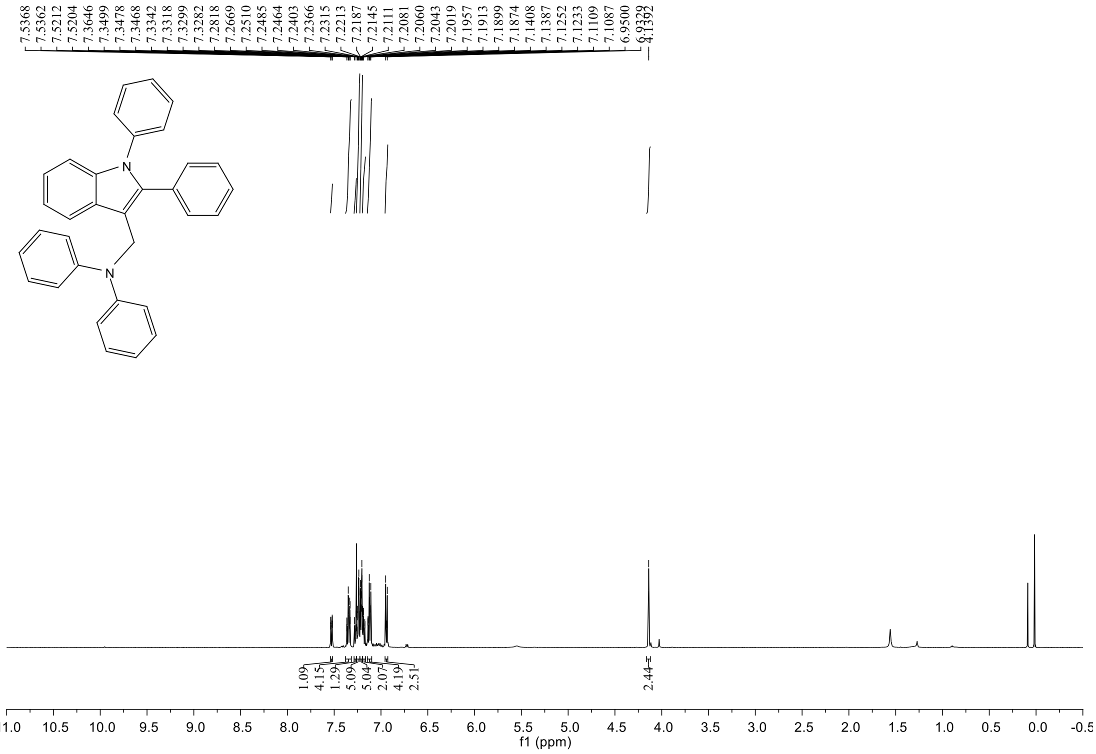

N-((1,2-diphenyl-1H-indol-3-yl)methyl)-N-phenylaniline(5e)

^{13}C-NMR

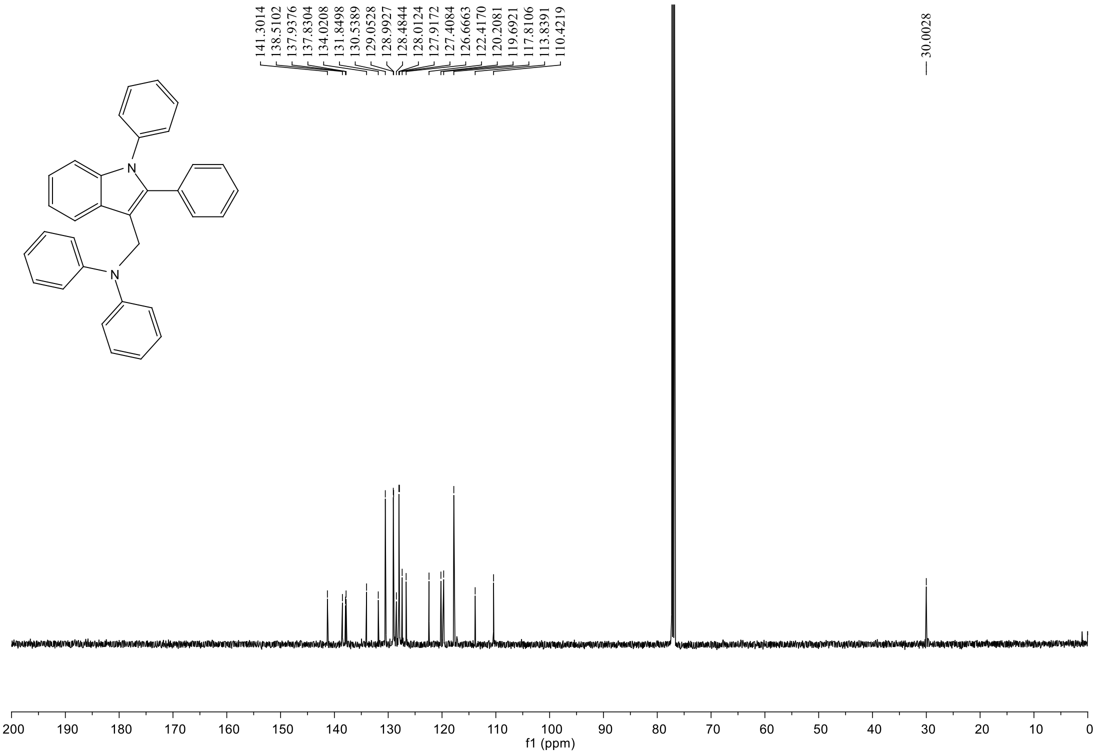

1,2-diphenyl-3-propyl-1H-indole (9a)

^{1}H NMR

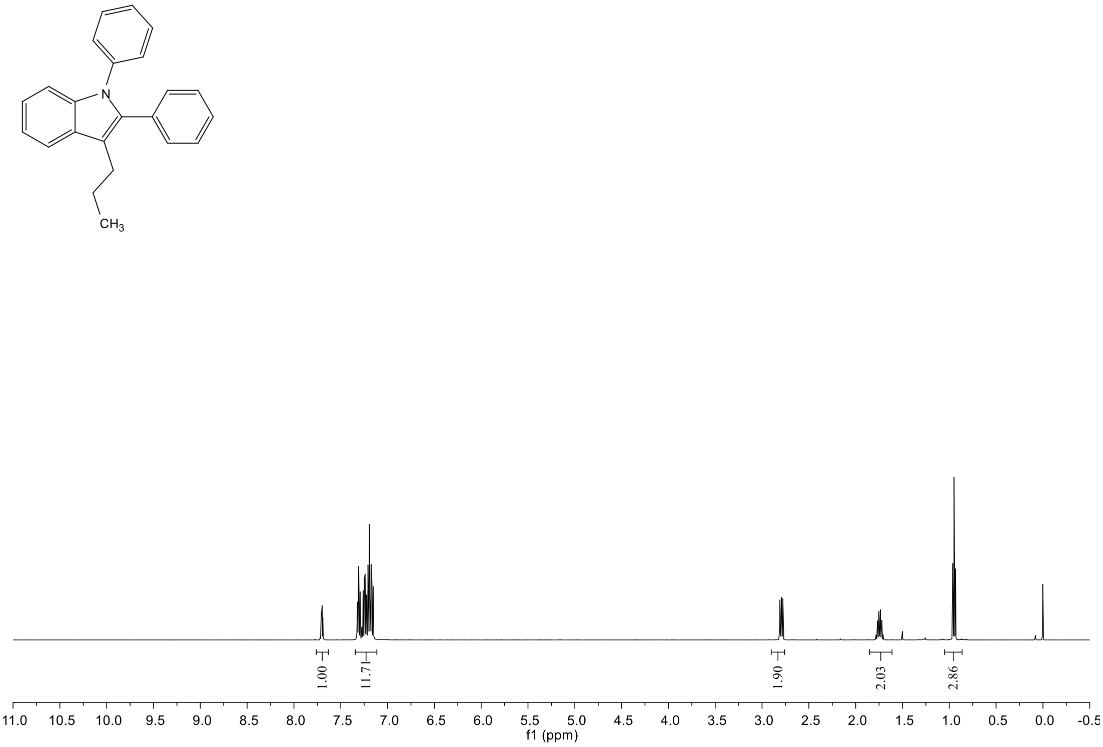

1,2-diphenyl-3-propyl-1H-indole (9a)

^{13}C-NMR

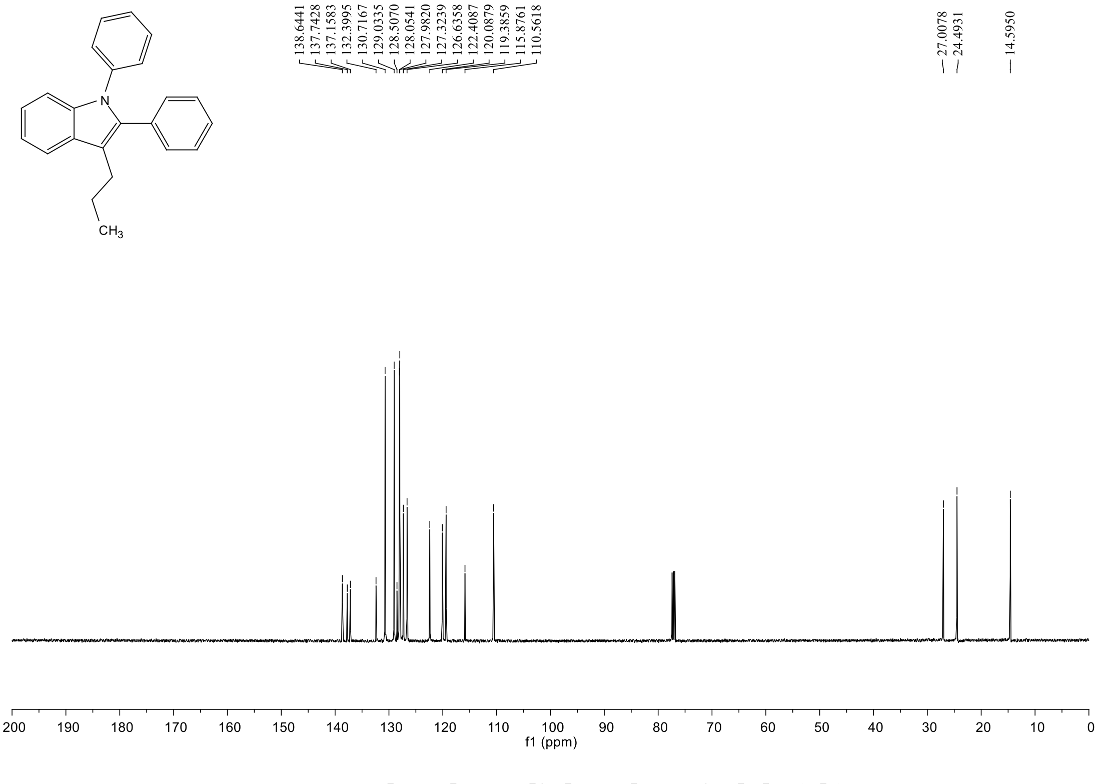

3-butyl-1,2-diphenyl-1H-indole (9b)

^{1}H NMR

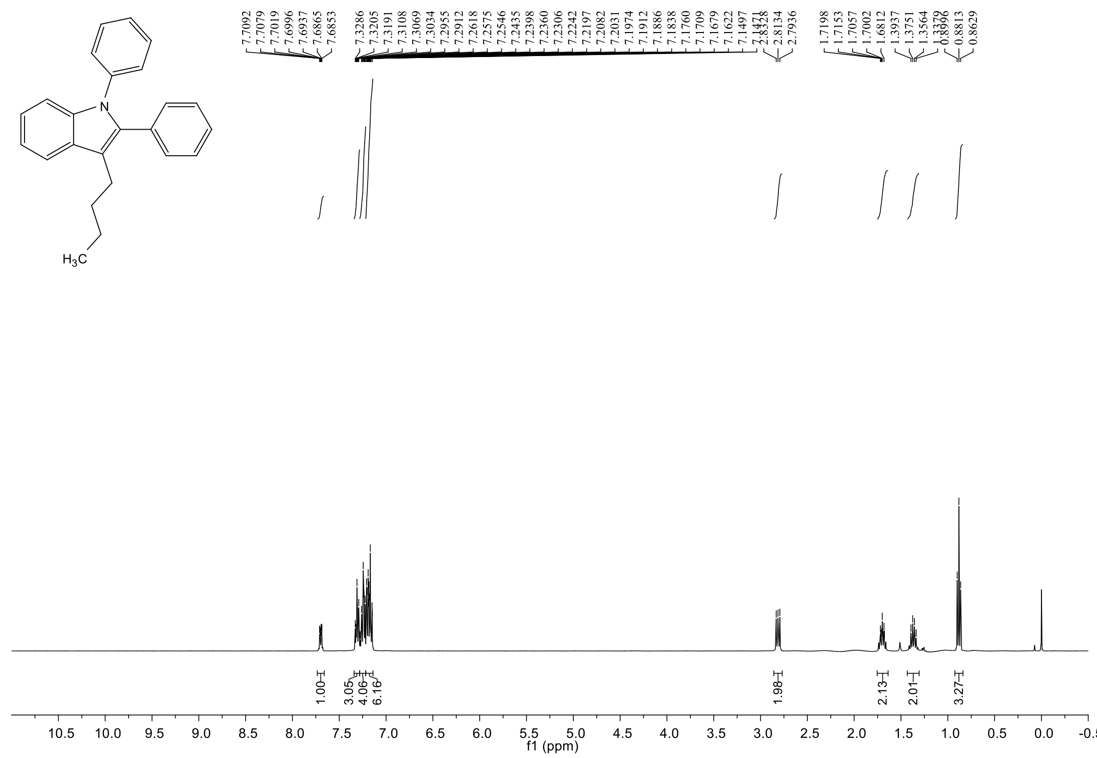

3-butyl-1,2-diphenyl-1H-indole (9b)

^{13}C-NMR

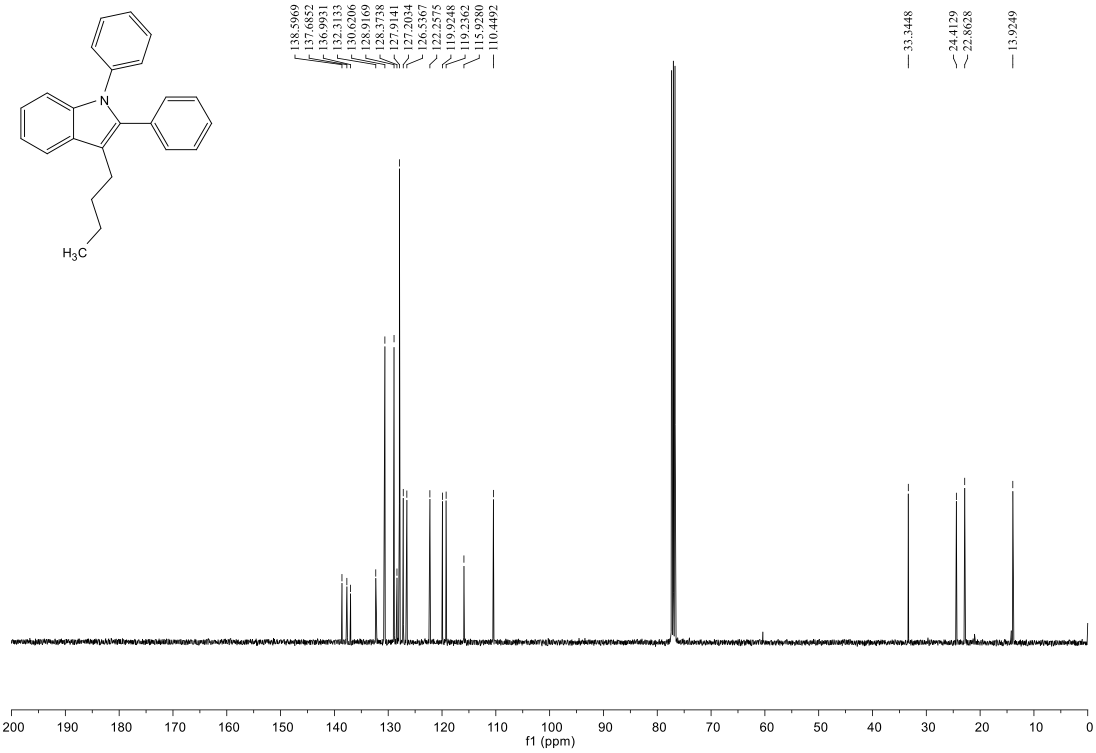

3-hexyl-1,2-diphenyl-1H-indole(9c)

1H NMR

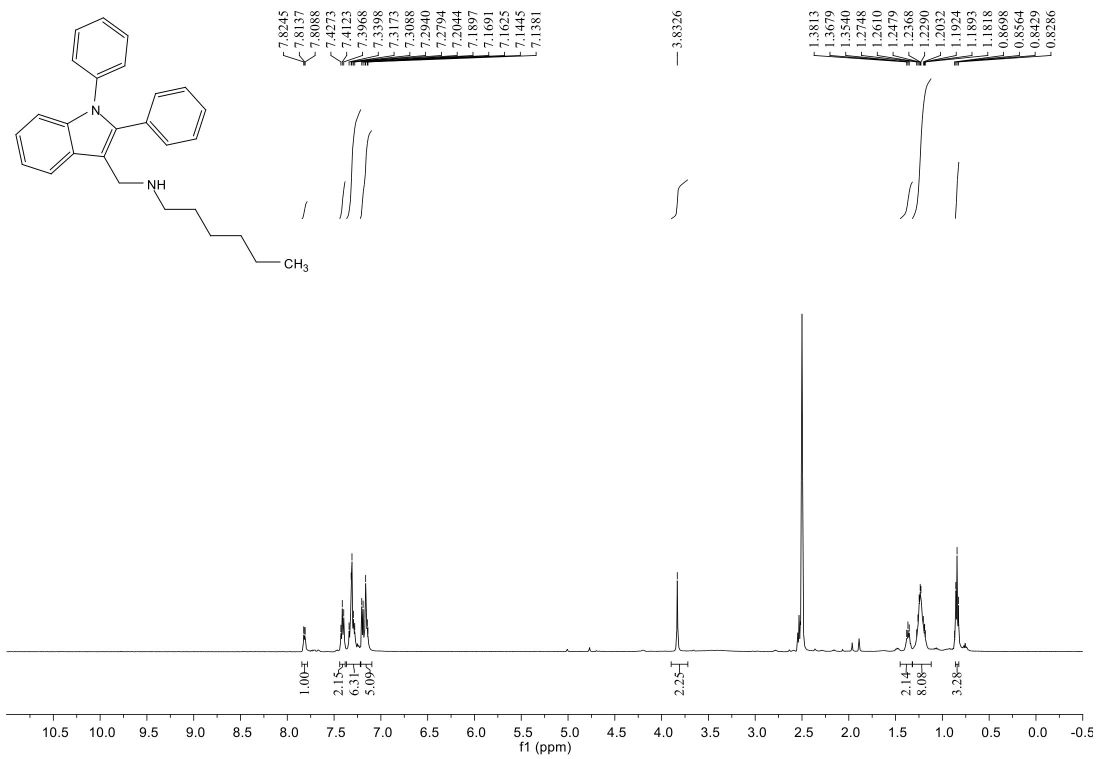

3-hexyl-1,2-diphenyl-1H-indole(9c)

13C-NMR

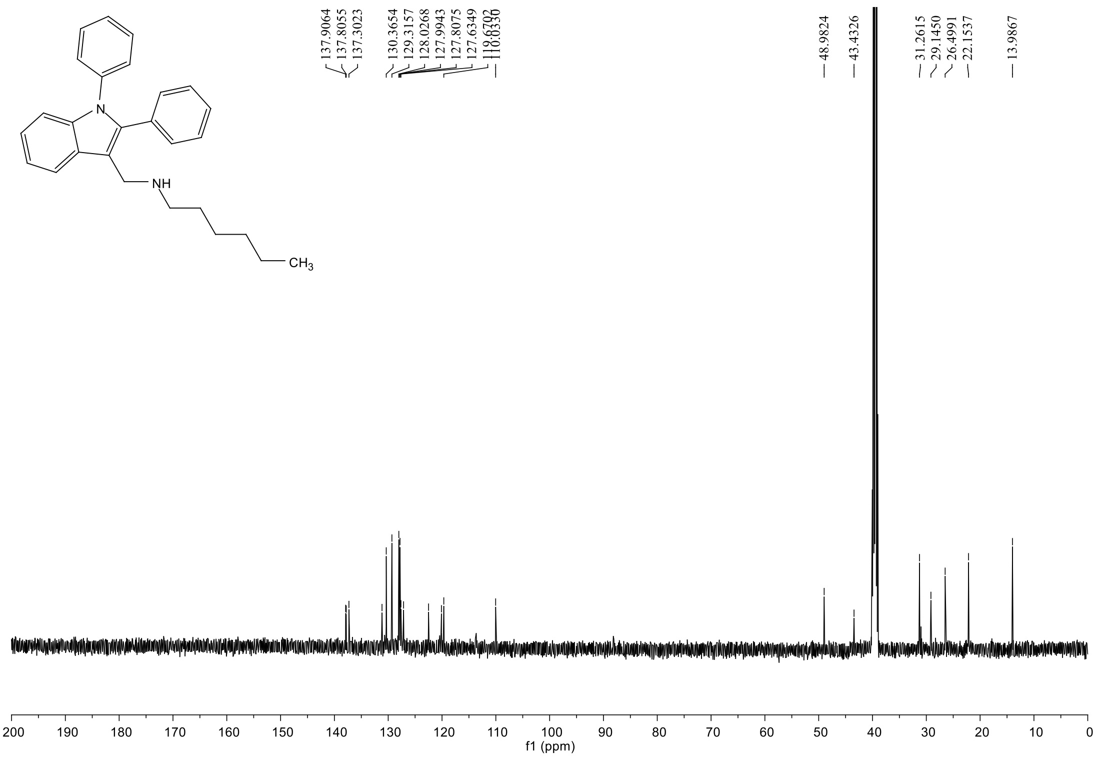

3-octyl-1,2-diphenyl-1H-indole(9d)

^{1}H NMR

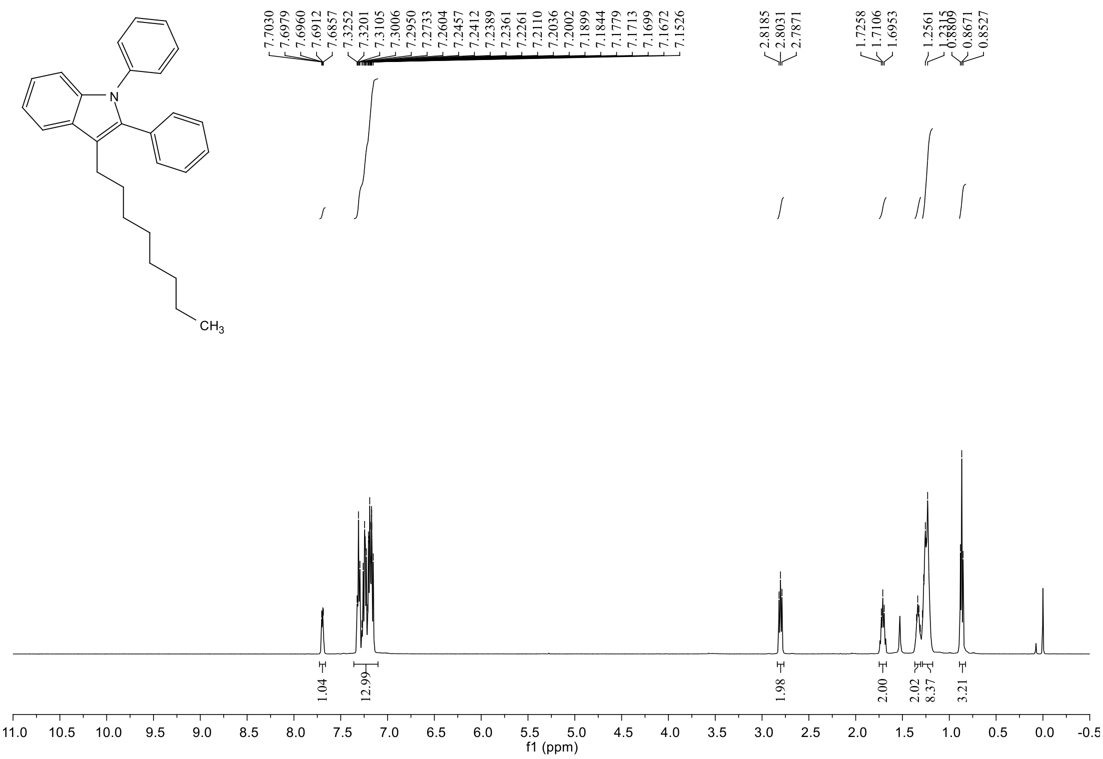

3-octyl-1,2-diphenyl-1H-indole(9d)

^{13}C-NMR

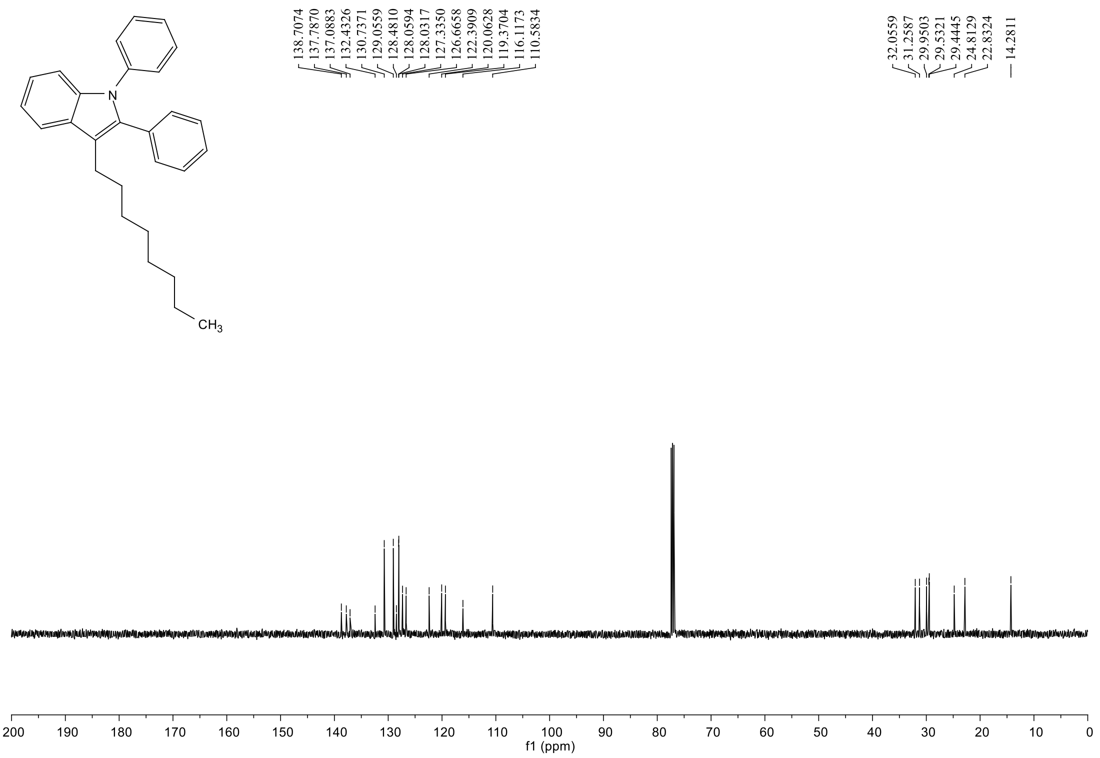

3-decyl-1,2-diphenyl-1H-indole (9e)

^{1}H NMR

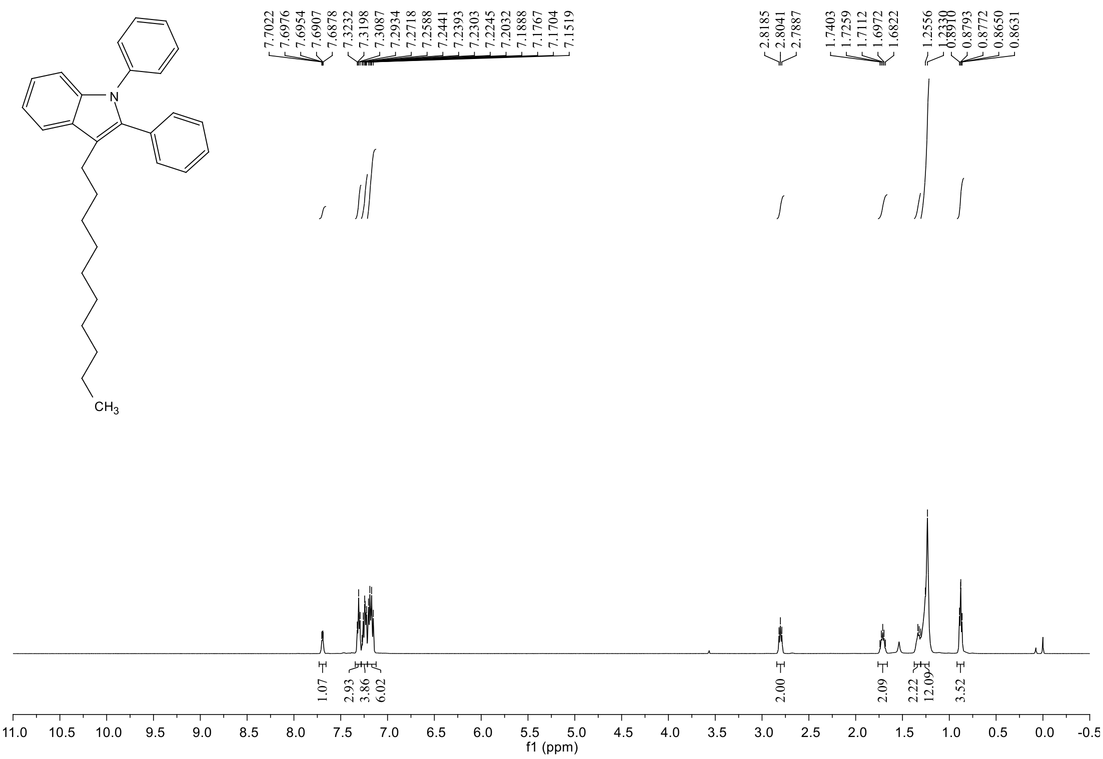

3-decyl-1,2-diphenyl-1H-indole (9e)

^{13}C-NMR

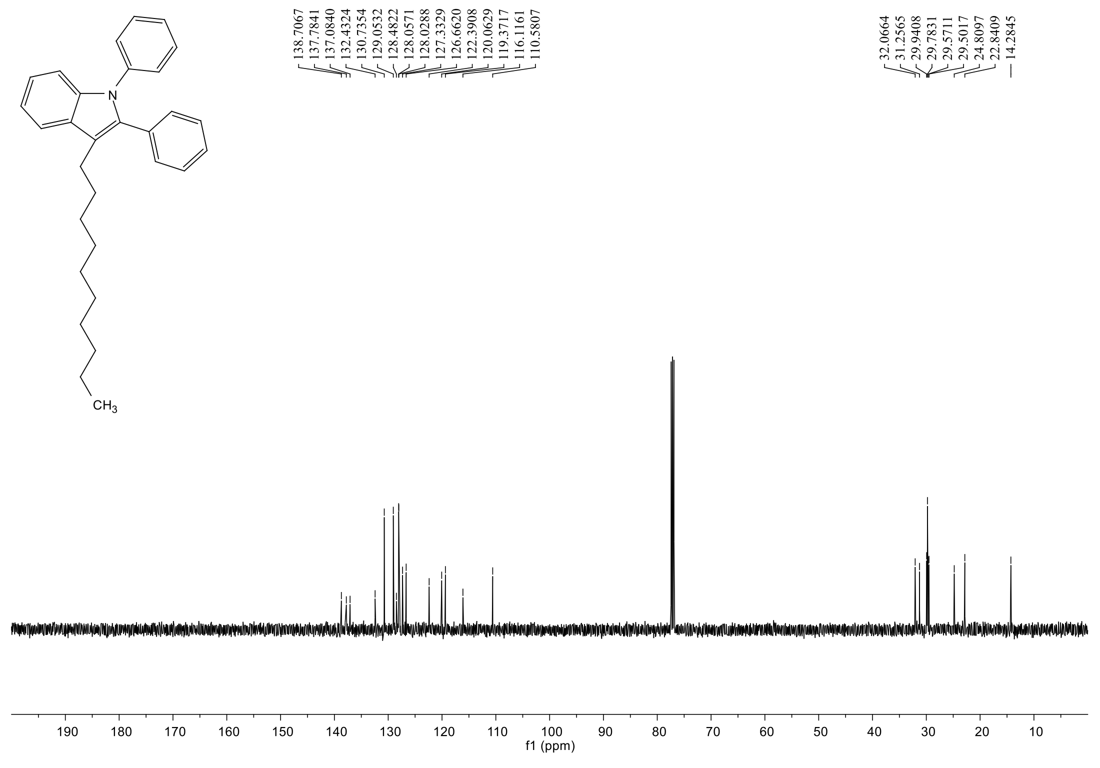

3-hexadecyl-1,2-diphenyl-1H-indole (9f)

^{1}H NMR

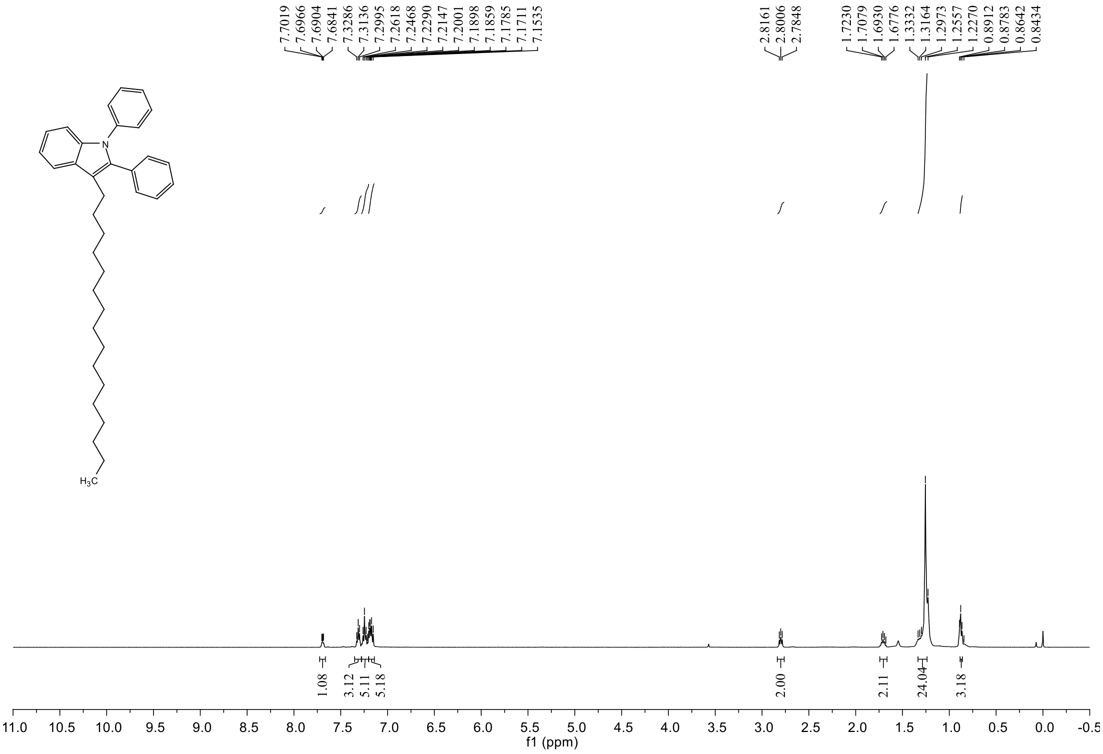

3-hexadecyl-1,2-diphenyl-1H-indole (9f)

^{13}C-NMR

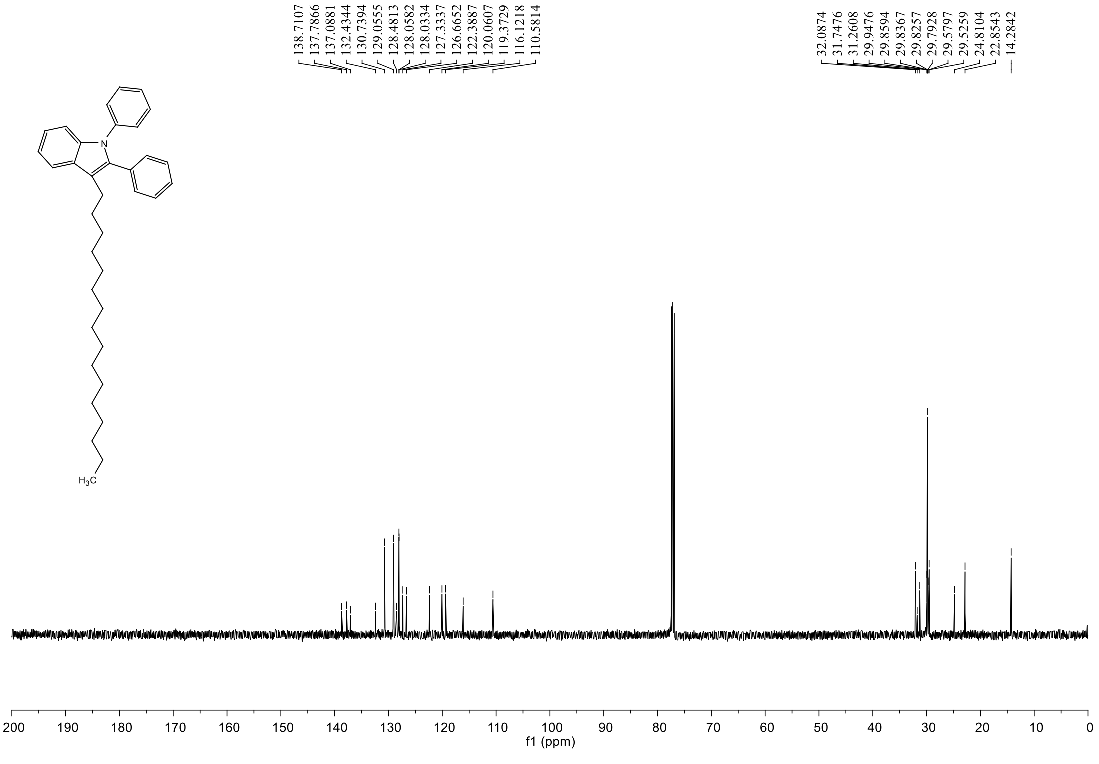

3-ethyl-1-phenyl-1H-indole (12a)

^{1}H NMR

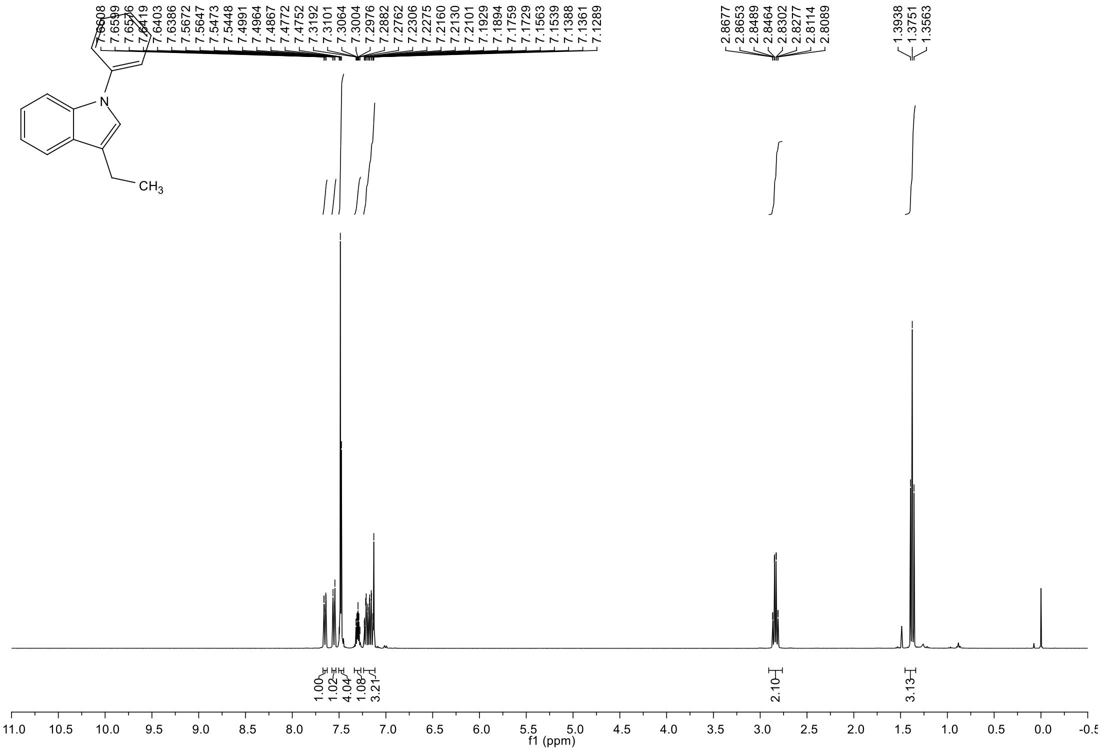

3-ethyl-1-phenyl-1H-indole (12a)

^{13}C-NMR

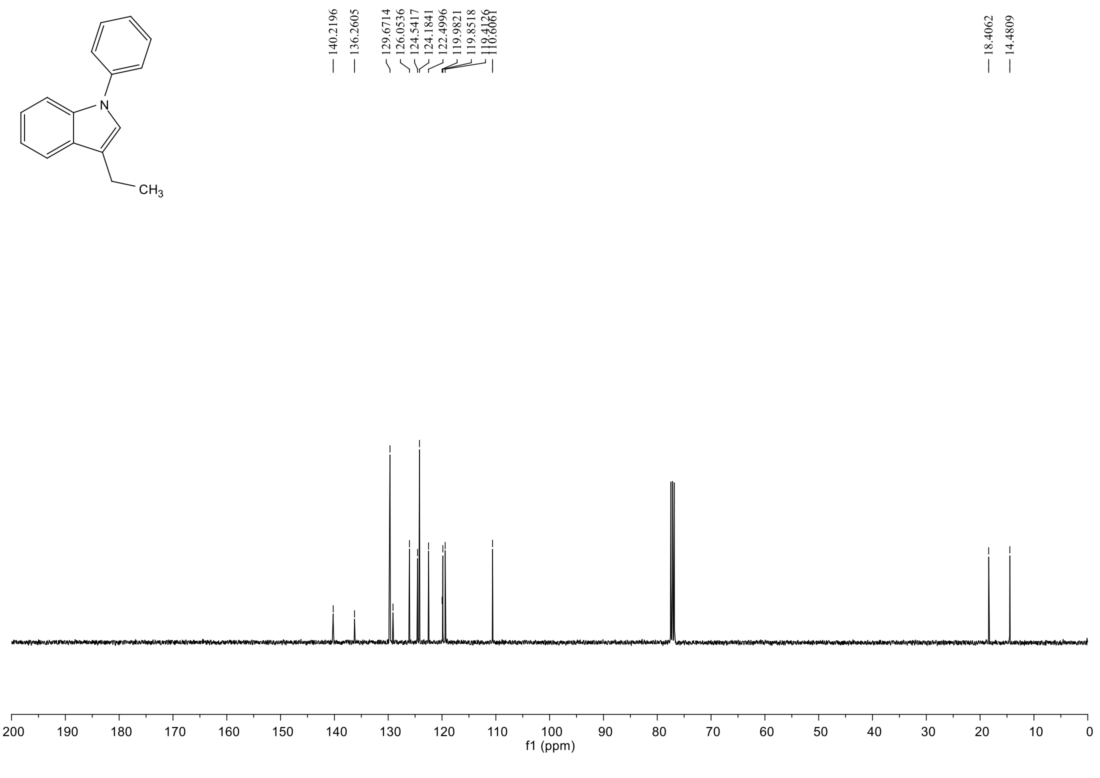

1-phenyl-3-propyl-1H-indole(12b)

^{1}H NMR

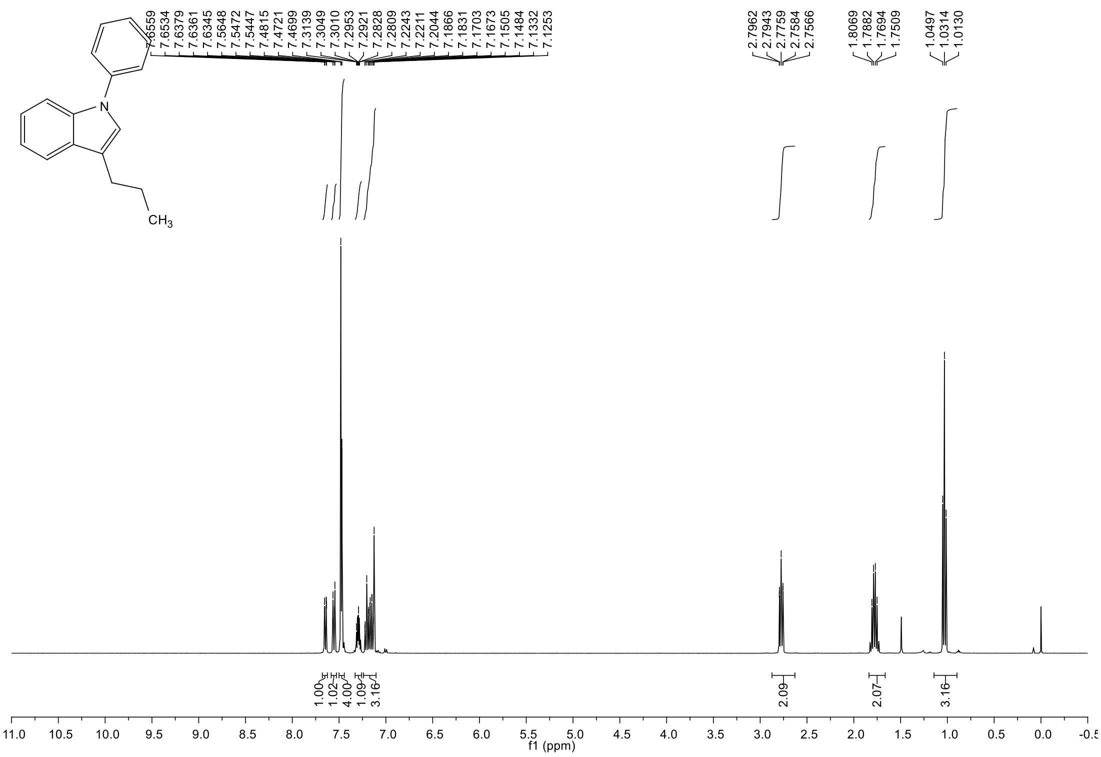

1-phenyl-3-propyl-1H-indole(12b)

^{13}C-NMR

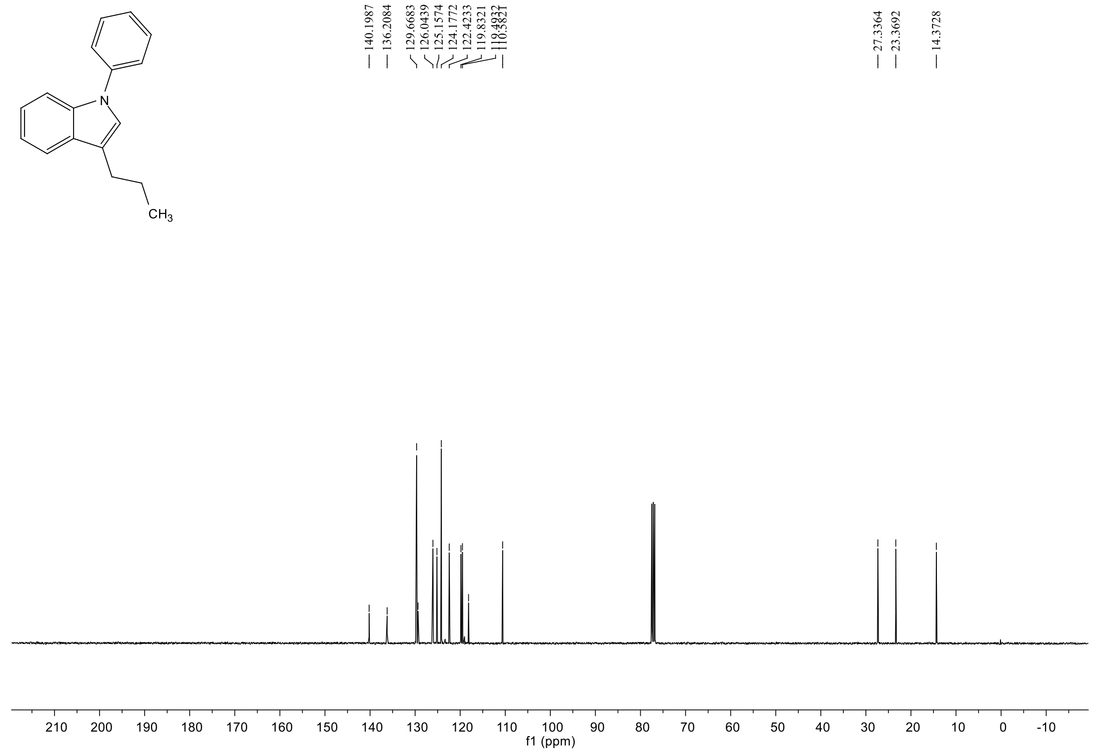

2-(1-phenyl-1H-indol-3-yl)ethanamine (12c)

^{1}H NMR

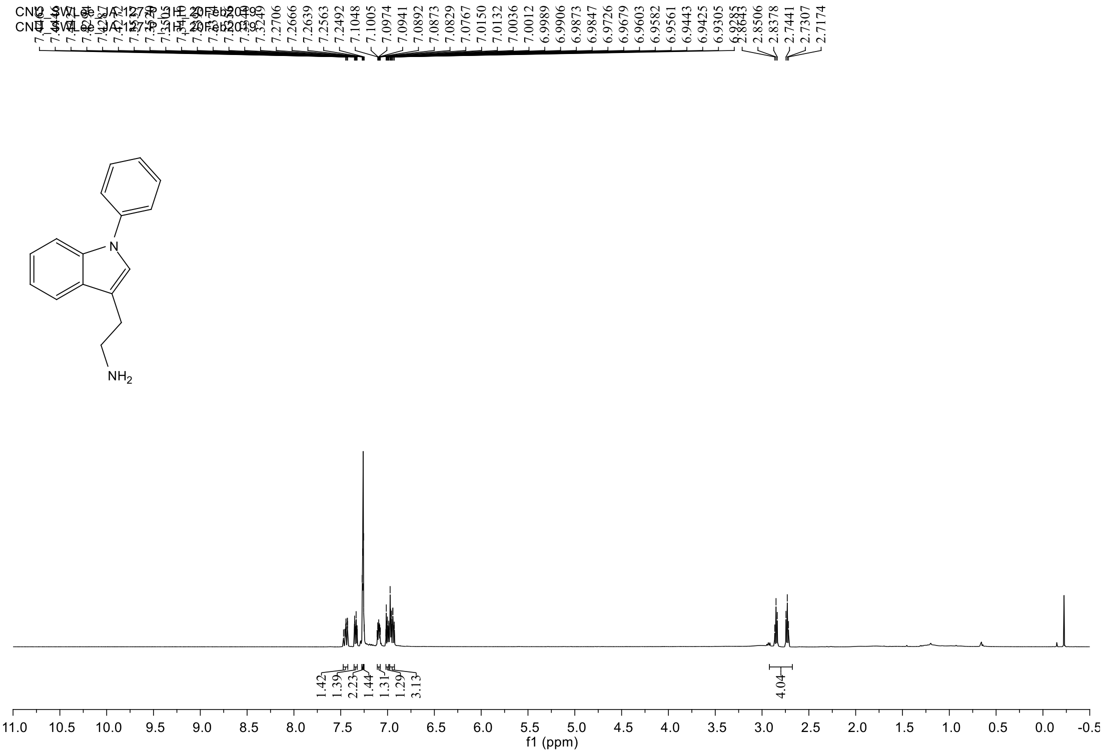

2-(1-phenyl-1H-indol-3-yl)ethanamine (12c)

^{13}C-NMR

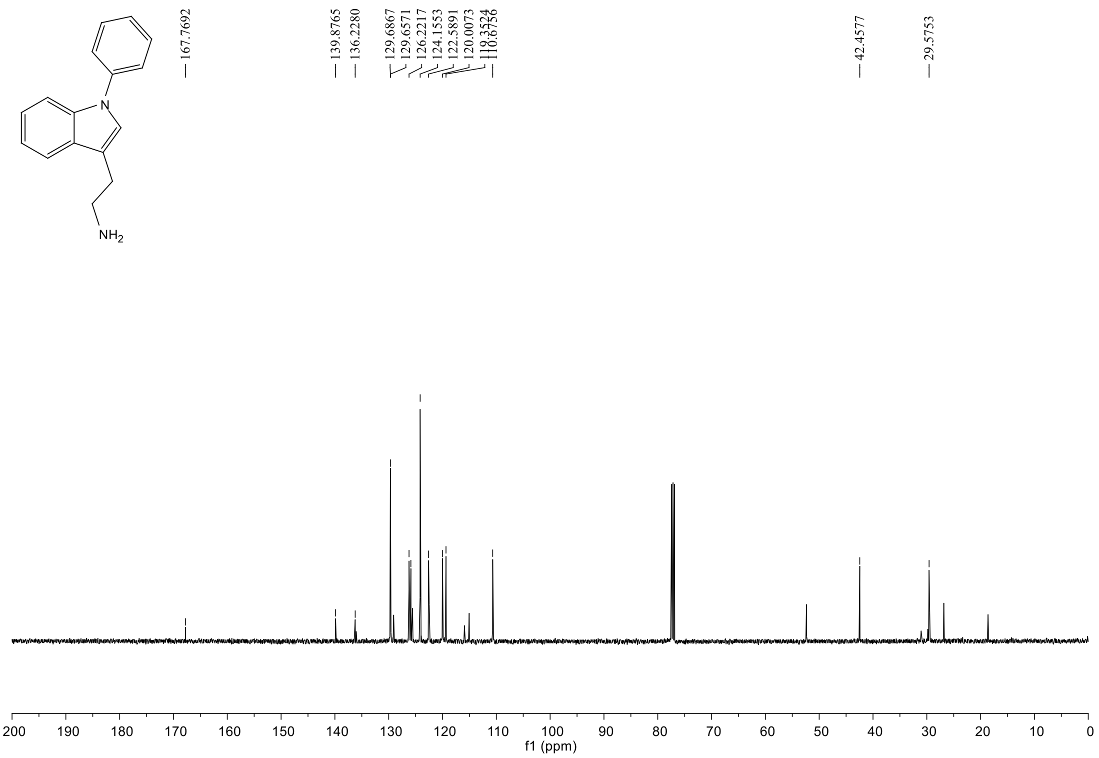

Supplement: Supplementary file 1 [file molecules-27-00899-s001.zip › molecules-1566435-supplementary.pdf]
